# Transcriptome Analysis of an Insecticide Resistant Housefly Strain: Insights about SNPs and Regulatory Elements in Cytochrome P450 Genes

**DOI:** 10.1371/journal.pone.0151434

**Published:** 2016-03-28

**Authors:** Khalid Mahmood, Dorte H. Højland, Torben Asp, Michael Kristensen

**Affiliations:** 1 Department of Agroecology, Aarhus University, Slagelse, Denmark; 2 Department of Molecular Biology and Genetics, Aarhus University, Slagelse, Denmark; Institute of Zoology, Chinese Academy of Sciences, CHINA

## Abstract

**Background:**

Insecticide resistance in the housefly, *Musca domestica*, has been investigated for more than 60 years. It will enter a new era after the recent publication of the housefly genome and the development of multiple next generation sequencing technologies. The genetic background of the xenobiotic response can now be investigated in greater detail. Here, we investigate the 454-pyrosequencing transcriptome of the spinosad-resistant 791spin strain in relation to the housefly genome with focus on P450 genes.

**Results:**

The *de novo* assembly of clean reads gave 35,834 contigs consisting of 21,780 sequences of the spinosad resistant strain. The 3,648 sequences were annotated with an enzyme code EC number and were mapped to 124 KEGG pathways with metabolic processes as most highly represented pathway. One hundred and twenty contigs were annotated as P450s covering 44 different P450 genes of housefly. Eight differentially expressed P450s genes were identified and investigated for SNPs, CpG islands and common regulatory motifs in promoter and coding regions. Functional annotation clustering of metabolic related genes and motif analysis of P450s revealed their association with epigenetic, transcription and gene expression related functions. The sequence variation analysis resulted in 12 SNPs and eight of them found in *cyp6d1*. There is variation in location, size and frequency of CpG islands and specific motifs were also identified in these P450s. Moreover, identified motifs were associated to GO terms and transcription factors using bioinformatic tools.

**Conclusion:**

Transcriptome data of a spinosad resistant strain provide together with genome data fundamental support for future research to understand evolution of resistance in houseflies. Here, we report for the first time the SNPs, CpG islands and common regulatory motifs in differentially expressed P450s. Taken together our findings will serve as a stepping stone to advance understanding of the mechanism and role of P450s in xenobiotic detoxification.

## Introduction

Insecticide resistance represents an important example of microevolution by natural selection and has become one of the major driving forces through altering the development of integrated pest management programs worldwide. Resistance to chemical insecticides in insects is mostly the consequence of either a change in the sensitivity of insecticide targets in the nervous system or by increased metabolism of insecticides before they reach their targets [1–3]. Metabolic-based resistance may result from two distinct, but additive genetic events: i) mutation of the enzyme leading to a better metabolism of the insecticide, and/or ii) mutation in a regulatory region leading to over-production of the metabolizing enzyme [3, 4]. These enzymes belong to gene families of esterases (EST), P450s monooxygenases (P450s), glutathione-S-transferases (GSTs) and ATP binding cassette transporter (ABCs) [5–9].

The housefly, *Musca domestica*, is a major pest, which can serve as a mechanical carrier of many pathogens emphasizing its significance as a pest. Houseflies have a preference for manure and other ‘filthy’ living conditions, making them carriers for transmission of diseases such as salmonellosis, typhoid fever, cholera and infantile diarrhea [10–12]. The housefly is ubiquitous and control is achieved through environmental sanitation, resource reduction and primarily through insecticides. Chemical insecticides normally work on the nerve system, where they either inhibit acetylcholine esterase (AChE) or directly work on the nerve receptors. The use of insecticides, though effective, also causes undesirable effects, which include resistance, elimination of non-target organisms, environmental damage and harm to human health, depending on the insecticide in question [13–17]. By understanding the mode of action of insecticides, and identifying the genetic mechanisms and mutations that causes resistance, scientists will ultimately enable early detection of resistance alleles in the field and help to improve management strategies. Bioassays have frequently been used to examine the resistance level of Danish field populations to insecticides [15, 16, 18]. The presence of metabolic-based resistance mechanisms was investigated by exposing flies to synergists prior to bioassays with insecticides and by measuring enzyme activities of each detoxification enzyme family [15–18]. At the molecular level, the frequency of the target-site *kdr* mutation has been investigated [19] and quantitative real-time RT-PCR was used to identify detoxification genes putatively involved in metabolic resistance [20, 21].

Spinosad is a relatively new insecticide and is a fermentation metabolite from the soil-borne actinomycete bacterium *Sacharopolyspora spinosad*, which acts on the nicotinic acetylcholine receptor in insects for effective control [22, 23]. It belongs to the group of spinosyns and is effective in control of lepidopterous and dipterous pests among others [22, 23]. Unfortunately, not long after its introduction, several cases of spinosad resistance have been reported worldwide [24]. It is believed that the resistance mechanism against spinosad involves both target-site and metabolic resistance [25]. Before spinosad was introduced in Denmark for housefly control, a baseline for spinosad toxicity was established using multiple Danish field strains [16]. The 791spin is the spinosad-selected strain derived from 791a, a laboratory strain derived from a multi-resistant field-collected sample of houseflies. The 791a strain proved highly resistant to pyrethroids and some anticholinesterases and showed some resistance to the chitin synthesis-disrupting larvicides [14, 16]. Resistance against neonicotinoids, such as imidacloprid and thiamethoxam, was also observed [21, 26, 27]. Low level resistance was observed for fipronil [17]. Selection with spinosad to create the 791spin strain caused a diminishment of the resistance towards fipronil, imidacloprid and thiamethoxam seen for the parental 791a strain. However, resistance towards synthetic pyrethroids and organophosphates was preserved. The female-specific spinosad resistance observed in the parental 791a strain was maintained in the selected strain. Furthermore, tests with the pesticide synergist piperonyl butoxide (PBO), which inhibits P450, indicated involvement of P450s in resistance of spinosad in the 791spin strain [27]. It is believed that spinosad resistance in 791spin is related to chromosome III, which is the location of the male-determining factor in this strain [28]. Several P450s of the CYP6 and CYP4 families have been suggested to be involved in spinosad resistance, by overexpression of genes in the resistant strain in comparison with the susceptible reference strain WHO-SRS [29]. But none has proven to be the sole contributor to resistance in this strain.

The recently published housefly genome [30] fueled us towards transcriptome analysis of our resistant housefly strain for investigation of metabolism-based insecticide resistance. For this purpose *de novo* assembly of a spinosad resistant housefly strain 791spin, where metabolism is believed to be the main resistance mechanism [29], was conducted. In the evolution of resistance, metabolic enzymes such as P450s are major weapons of houseflies and recently we reported differential expression of P450s in 791spin [29]. These P450s include *cyp4g2*, *cyp6g4*, *cyp6a1*, *cyp6a36*, *cyp6a37*, *cyp6d1*, *cyp6d3* and *cyp12a2* [29]. The constitutive over-expression and induction of P450s in insecticide resistant species are common phenomena that are responsible for detoxification of insecticides [31, 32]. Several studies suggested that over-expression is mediated through *trans* and/or *cis* regulatory factors[33–35]. However little is known about these regulatory components. It would be interesting to look for transcriptional elements in the promoter region, regulatory elements and epigenetic modification to understand molecular mechanism involved. Epigenetic modifications in insects are thought to be less significant initially but now it’s well known to play a role in insect [36–38]. Methylation of DNA occur in almost all eukaryotes, but varies a lot among taxa[39], and reportedly methylation in insects is CpG specific [38, 40]. Considering all these information and significance of our selected P450s, we analyzed them for regulatory elements to get deeper insights using the recently published housefly genome and availability of sophisticated bioinformatic tools. Here we report, for the first time, information about CpG islands, identification of novel single nucleotide polymorphisms (SNPs) in a resistant strain compared to a susceptible strain as well as specific regulatory motifs in above mentioned P450s. Furthermore, identified motifs were linked to probable transcription factors. Nonetheless, our analysis of resistant strain 791spin led us to understand expression and regulatory elements of transcription of selected P450s that can provide insights about microevolution of insecticide resistance mechanism.

## Materials and Methods

### Housefly strains and breeding

The spinosad selected 791spin strain was made by selection with spinosad of the multi-resistant 791a strain, which was collected in Denmark in 1997. The 791spin females were 21-fold resistant to spinosad at the LC_50_, whereas 791spin male houseflies were 6-fold resistant [27]. The flies were collected on private land with consent of the owner. The field collection did not involve endangered or protected species.

Housefly breeding followed standard laboratory conditions. Oviposition was performed on crumpled filter paper soaked in whole milk. Breeding jars (5 L plastic buckets) containing 4 L of medium were seeded with 200 mg of eggs, corresponding to 2,700 eggs. The breeding medium consisted of wheat bran 400 g, lucerne meal 200 g, baker’s yeast 10 g, malt extract 15 mL, whole milk 500 mL and water 500 mL. For adult feeding, cube sugar and water were given continuously. Feeding started after emergence with whole-milk powder mixed with icing sugar (1:1 w/w) [14]. Houseflies for transcriptome analysis were five to seven days old, adult male and female flies, which were fed sugar as the only food source.

### Transcriptome assembly, annotation and functional classification analysis

Total RNA from whole bodies of pooled flies (*ca*. 1.2 g equivalent to 60 flies) was extracted using the RNeasy Maxi Kit (Qiagen). Flies were thoroughly grounded with liquid nitrogen, a mortar and pestle and otherwise following the manufacturer’s protocol. Isolated RNA was DNase-treated and concentrated using the RNeasy MinElute Kit (Qiagen). Gel electrophoresis and spectrophotometry (Nanodrop; NanoDrop Technologies, Wilmington, USA) was performed to assess the integrity and the concentration of each RNA sample, which was dissolved in RNase-free water and stored at -20°C until use.

Normalization was performed using TRIMMER cDNA normalization kit (EVR_GEN) to decrease the prevalence of abundant transcripts before sequencing as described by [41]. A normalized cDNA library was prepared from 12.2 μg mRNA prepared from adult male and female houseflies [42]. The normalized cDNA library was size fractioned to approx. 500–1,200 bp. High throughput sequencing on GS FLX^++^ of the *Musca* cDNA library was done according to the standard protocols using a Genome Sequencer FLX Titanium Instrument (Roche Diagnostics). Preparation of cDNA, normalization and sequencing was performed in Eurofins MWG GmbH (Ebersberg, Germany).

Raw data generated from 454 pyrosequencing were preprocessed to remove low quality sequences including a) adapters that were added for reverse transcription and 454 sequencing, b) primers, c) very short (<40 bp) sequences, and d) low quality sequences using Newbler program. The preprocessed data were clustered and assembled in contigs using MIRA 4.0 along with GS *De novo* Assembler (Newbler v 2.6) supplied with the GS FLX Titanium sequencer and contigs were initially analyzed by BLAST analysis. Briefly describing, two different assembler programs were used a) Newbler 2.6 (Roche) and b) MIRA. During assembly primer sequences and poly-A tails were trimmed from raw reads. Two main assembly parameters for each program were used as a) minimum percentage identity 80–95% for each assembler and b) minimum overlap length of 20–40 bp for MIRA and 40–60 bp for Newbler. The “-cdna” mode was used for Newbler but also tested “–urt “option to improve contig formation in low depths portion of the assemblies.

Annotation of assembled sequences was carried out using BLASTX searches against the NCBI non-redundant protein sequence database in the Blast2GO program. Sequences that shared similarities with known protein sequences in BLASTX searches with significant similarity (E<1e^-10^) were identified using the online tool InterProScan 5.0. In order to analyze transcriptome data, two separate programs (Blast2GO and DAVID) were used to determine gene function and enrichment of certain functions. The Blast2GO program was used to assign Gene Ontology (GO) terms to the annotated sequences to predict the functions of the unique sequences and encoded translated proteins [43, 44]. Furthermore, GO term enrichments were performed through the online database program “Database for Annotation, Visualization and Integrated Discovery (DAVID)” using the Expression Analysis Systemic Explorer (EASE)[45]. This program identifies significantly enriched terms (based on GO terms) and classified them as functional groups that are enriched in a given transcriptome. DAVID cluster genes into functional groups using a Fisher’s exact test to identify significantly enriched functional groups. DAVID was run with *p*-value < 0.05 to select annotation clusters, which was considered significantly enriched. Moreover, Kyoto Encyclopedia of Genes and Genomes (KEGG) analysis was used to identify potential pathways represented in the transcriptome [46–48].

### SNPs analysis of selected-differentially-expressed P450s

The sequence fragments of eight P450s from the resistant 791spin strain were compared to respective sequences of the susceptible reference strain WHO-SRS. These eight P450s were *cyp4g2* (NM_001286897), *cyp6g4* (NM_001286882), *cyp6a1* (NM_001287230), *cyp6a36* (XM_005184332), *cyp6a37* (XM_005184336), *cyp6d1* (XM_005184130), *cyp6d3* (NM_001286881) and *cyp12a2* (XM_005179997). The *cyp4g2* has 14 different contigs from the resistant strain (S1 Table). The *cyp6g4* has three contigs from the resistant strain 791spin. For SNPs analysis for *cyp6a1*, we used four contigs from 791spin and a sequence from a phenobarbital resistant strain from USA [49]. Three contigs from 791spin and a sequence from the pyrethroid resistant strain ALHF from USA [50] of *cyp6a36* were aligned together with susceptible strain WHO-SRS for SNPs identification. For this purpose, *cyp6a37* has five contigs from the resistant strain 791spin and a sequence from the pyrethroid resistant ALHF strain from USA [50]. The *cyp6d1* has five contigs from strains 791spin and a sequence from the pyrethroid resistant LPR strain from USA [51], whereas *cyp6d3* has two contigs from resistant strain 791spin and a strain from USA [52] used for SNPs analysis. Similarly, for *cyp12a2*, eight contigs from the spinosad resistant strain 791spin were aligned together to identify SNPs (S1 Table). All sequences of each gene were aligned with their respective sequence from the reference strain as consensus reference by Clustal W method using the *Jalview* alignment tool. We reported SNPs when consensus base ratio was 1.0. The consensus base ratio is the number of contigs or sequences derived from a single source mapped to a reference sequence having a nucleotide that differs from the corresponding nucleotide in the reference sequence. If the ratio was less than 1.0, some contigs or sequences have the same nucleotide as the reference sequence (susceptible) and is not considered as SNPs due to less confidence. Thus, a highly stringent criterion in imposed to identify SNPs.

### Discovery of DNA regulatory motifs

DNA motifs were searched in the promoters of differentially co-expressed P450 genes using the MEME (Multiple Em for Motif Elicitation) tool, which is a tool for discovering motifs in a group of related DNA or protein sequences [53]. A motif is a sequence pattern that occurs repeatedly in a group of related proteins or DNA sequences. MEME represents motifs as position-dependent letter-probability matrices, which describe the probability of each possible letter at each position in the pattern. Individual MEME motifs do not contain gaps. Patterns with variable-length gaps are split by MEME into two or more separate motifs [54]. All promoter sequences were extracted from 1,000 bp upstream regions of the P450 genes from the housefly genome with the filter that these sequences should not contain any unknown nucleotides (N) along with gene orientation taken into account. Similarly, mRNA sequences were extracted and checked for common motifs. The MEME analysis has been run with the following settings; the motifs should have a length between 6 and 50 nucleotides, the distribution model used was the default Zero Or One Per Sequence (ZOOPS).

The motifs identified using MEME were analyzed through GOMO (Gene Ontology for MOtifs) [55]. The purpose of GOMO is to identify possible roles (Gene Ontology terms) for DNA binding motifs. GOMO takes a motif and scores the upstream (promoter) region of each gene in the selected organism according to its binding affinity for the motif. Using these scores and the GO annotations of the organism's genes, GOMO determines which GO terms are associated with the (putative) target genes of the binding motif [55]. GOMO was run using the “single species category” which gave access to *Drosophila melanogaster* database. There is a high level of synteny between *M*. *domestica* and *D*. *melanogaster* and the ortholog counterparts of the *Musca* P450s in this study where selected in the *Drosophila* database, which contain a high level of functional knowledge of motifs and transcription factors. Similarly, TOMTOM has been employed on the significant motifs found by MEME to find a known transcription factor [56]. The TOMTOM is a tool for comparing a DNA motif to a database of known motifs, and hits provide information on the number of known transcription factors to which this motif is sequence-wise close using Pearson correlation coefficient with E-value <10. TOMTOM was run using the default setting, thus searched motif in all *Drosophila* databases. The *p*-value is the probability that the match occurred by random chance according to the null model, the E-value is the expected number of false positives in the matches up to this point and the q-value is the minimum False Discovery Rate (FDR) required to include the match.

## Results

### Sequencing and assembly

**Fig 1:**
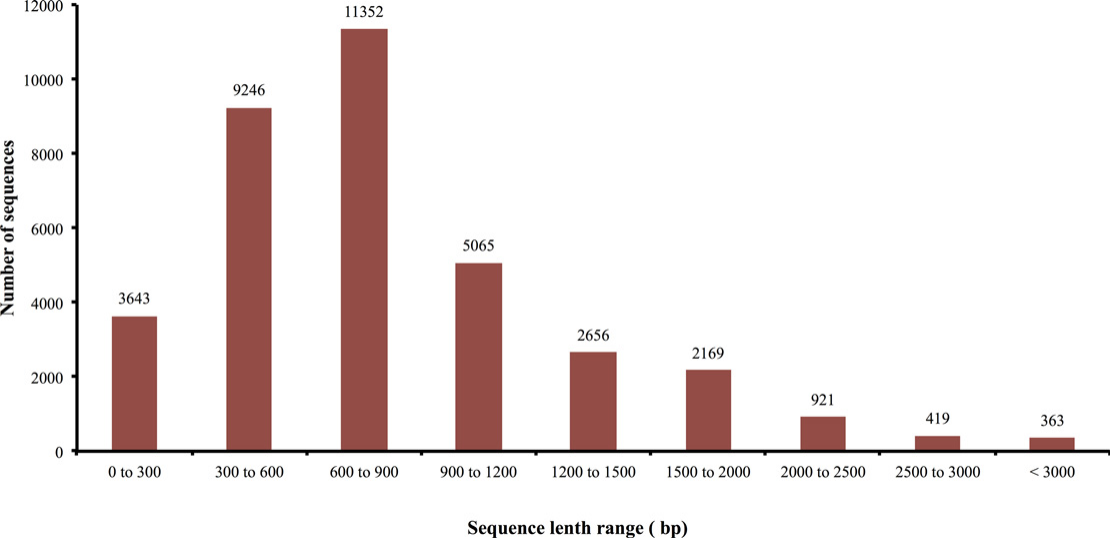
Length distribution of the assembled contigs.

The normalized cDNA library was prepared from a pool of houseflies of mixed sex for Roche 454 pyrosequencing. The sequencing generated 666,537 reads consisting of 315,617,305 bp and average sequence length was 473 bp. After removal of adaptor sequences, data were aligned and *de novo* assembled using version 2.6 of the newbler assembler (454 Life Sciences/Roche, Branford, CT) into 387,594 clean reads (58.1%) while 3,446 reads were too short. The *de novo* assembly of clean reads gave 35,834 contigs consisting of 30,645,588 bases. The contigs length varied from 40 bp to 6,150 bp with an average length of 855 bp. The summary of sequencing and assembly is shown in Table 1. The size distribution of the reads in contigs is shown in Fig 1. The raw reads from this library has been deposited in GenBank with the Accession Number: GSE65891 (http://www.ncbi.nlm.nih.gov/geo/query/acc.cgi?acc=GSE65891).

**Table 1:** Summary of run statistics and assembly.

Large run results	
Total number of reads	666,537
Total number of bases w/o keys, tags and bad quality bases	315,617,305
Average read length w/o keys, tags and bad quality bases	473
Assembly results	
Number assembled	387,594
Number too short	3,446
Sum of large contigs	
Total number of reads	243,685
Number of large contigs	8,061
Total number of bases	13,356,970
N50	1,679
Sum of all contigs	
Total number of reads	387,594
Number of all contigs	35,834
Total number of bases	30,645,588
Average contig length	855
Shortest contig length	40
Longest contig length	6,150
N50	986

### Annotation and gene ontology analysis

Annotations of assembled sequences were carried out by BLASTx against the NCBI non-redundant protein sequence databases using the software Blast2GO [43, 44]. A total of 21,780 sequences (61%, with cutoff *e*-value <1e^-5^) were homologous to proteins in the non-redundant database. We identified 20,460 sequences (93.9%) that shared significant similarities (E-value ≤1e^-11^) with known protein sequences and 1,320 sequences (6.1%), which shared weak similarity with an *e*-value <1e^-6^ to 1e^-10^. Further analysis of BLAST data indicated that more than a third of the hits had an *e*-value < 1e^-100^ (S1 Fig).

**Fig 2:**
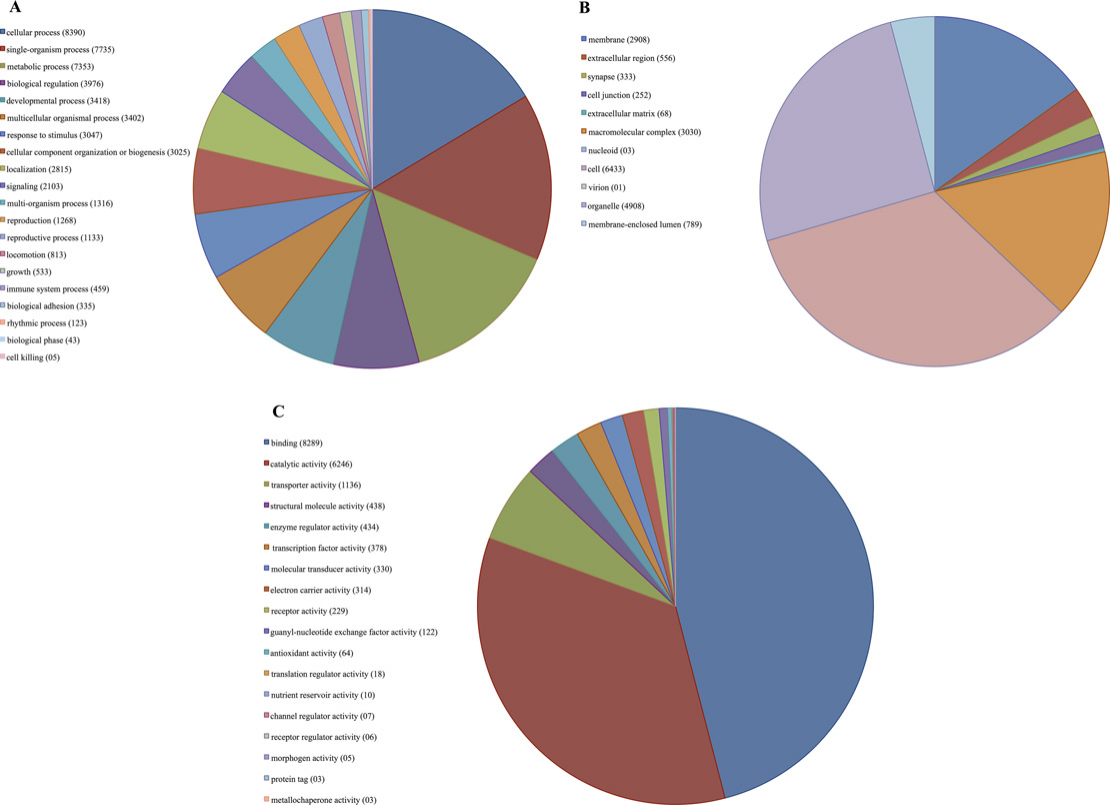
Distributions of the annotated sequences in three GO categories (Level Two). A: biological process, B: Cellular component, C: Molecular function.

Most sequence hits were to *M*. *domestica* (88.38%), followed by *Ceratitis capitata* (2.23%) and *Drosophila melanogaster* (1.23%, S2 Fig). The top 10 organisms were all Diptera species, with the exception of the parasitic roundworm, *Ancylostoma ceylanicum* (S2 Fig). The Blast2GO program was also used to assign Gene Ontology (GO) terms to the annotated sequences to predict the functions of the unique sequences and encoded translated proteins; whereas DAVID examining the enriched GO terms and clustering the similar GO terms based on their significance in resistant housefly strain. In the Blast2GO analysis, a total of 9,003, 13,030 and 9,349 sequences were assigned to biological processes (BP), molecular functions (MF) and cellular components (CC) GO categories, respectively. The distribution of the annotated sequences in these three GO categories (broad level 2 terms) was shown in Fig 2. The highest number of annotated transcripts by BP was associated with cellular process (16%), single organism process (15%) and metabolic process (14%) (Fig 2A). In the CC category, cell (33%), organelle (25%) and macromolecular complex (16%) were the three most abundant categories while in the molecular function GO category, the sequences were predominantly assigned to binding (46%), catalytic activity (35%) and transport activity (6%) (Fig 2B and 2C).

The DAVID analysis identifies significant enriched terms and arranges GO terms into functional clustering based on significance. In the BP category, most significant enriched terms in the transcriptome of resistant housefly were cellular component organization with 155 genes, cellular processes with 331 genes and regulation of metabolic processes with 109 genes (Table 2). In the MF category, protein binding (308 genes), transcription activator activity (16 genes) and hydrolase activity (50 genes) were significantly enriched terms (Table 2). Similarly, in the CC category, protein complex (133 genes), nuclear part (83 genes) and macromolecular complex (158 genes) were the significantly enriched terms (Table 2).

**Table 2:** Over-represented GO terms from resistant housefly transcriptome. GO term enrichment were performed using Expression Analysis Systematic Explorer (EASE) implemented in the Database for Annotation, Visualization, and Integrated Discovery (DAVID 6.7).

Term	Count	*P*-Value	Bonferroni	Benjamini	FDR
Biological Processes (BP)					
GO:0016043~cellular component organization	155	5.59E-14	1.07E-10	1.07E-10	9.56E-11
GO:0009987~cellular process	331	6.12E-12	1.17E-08	5.87E-09	1.05E-08
GO:0019222~regulation of metabolic process	109	2.24E-10	4.30E-07	1.43E-07	3.84E-07
GO:0065007~biological regulation	191	1.21E-09	2.31E-06	5.79E-07	2.06E-06
GO:0006996~organelle organization	91	1.69E-09	3.25E-06	6.50E-07	2.90E-06
GO:0050789~regulation of biological process	175	7.14E-09	1.37E-05	2.28E-06	1.22E-05
GO:0060255~regulation of macromolecule metabolic process	98	8.29E-09	1.59E-05	2.27E-06	1.42E-05
GO:0031323~regulation of cellular metabolic process	94	4.96E-08	9.53E-05	1.19E-05	8.49E-05
GO:0051179~localization	132	9.34E-08	1.79E-04	1.99E-05	1.60E-04
GO:0050794~regulation of cellular process	160	1.93E-07	3.71E-04	3.71E-05	3.30E-04
GO:0010468~regulation of gene expression	86	2.49E-07	4.78E-04	4.35E-05	4.26E-04
GO:0033036~macromolecule localization	54	2.98E-07	5.71E-04	4.76E-05	5.09E-04
GO:0051234~establishment of localization	114	3.35E-07	6.43E-04	4.95E-05	5.73E-04
GO:0034641~cellular nitrogen compound metabolic process	110	4.46E-07	8.57E-04	6.12E-05	7.64E-04
GO:0007010~cytoskeleton organization	50	4.83E-07	9.27E-04	6.18E-05	8.26E-04
Molecular Function (MF)					
GO:0005515~protein binding	308	1.22E-06	7.86E-04	7.86E-04	0.0018253
GO:0005488~binding	430	4.34E-06	0.0027792	0.0013905	0.0064616
GO:0008092~cytoskeletal protein binding	28	1.05E-05	0.0067290	0.0022480	0.0156750
GO:0016563~transcription activator activity	16	4.96E-05	0.0313181	0.0079232	0.0738502
GO:0016818~hydrolase activity, acting on acid anhydrides, in phosphorus-containing anhydrides	50	2.01E-04	0.12109596	0.0254855	0.2992483
GO:0016817~hydrolase activity, acting on acid anhydrides	50	2.21E-04	0.13236004	0.0233853	0.3291028
GO:0016462~pyrophosphatase activity	49	2.77E-04	0.16303795	0.0251047	0.4123734
GO:0003779~actin binding	17	3.45E-04	0.19870473	0.0273108	0.5130181
GO:0000166~nucleotide binding	86	4.73E-04	0.26216982	0.0332181	0.7034381
GO:0017111~nucleoside-triphosphatase activity	47	6.60E-04	0.34563135	0.0415217	0.9798076
GO:0030528~transcription regulator activity	54	7.25E-04	0.37221362	0.0414400	1.0751055
GO:0032555~purine ribonucleotide binding	67	0.0015268	0.62505230	0.0784952	2.25187183
GO:0032553~ribonucleotide binding	67	0.0015268	0.62505230	0.0784952	2.25187183
GO:0008641~small protein activating enzyme activity	5	0.0021777	0.75331878	0.1020725	3.197491
GO:0000175~3'-5'-exoribonuclease activity	5	0.0021777	0.75331878	0.1020725	3.197491
Cellular Components (CC)					
GO:0043234~protein complex	133	2.56E-10	9.68E-08	9.68E-08	3.54E-07
GO:0044428~nuclear part	83	4.77E-10	1.80E-07	9.02E-08	6.60E-07
GO:0032991~macromolecular complex	158	6.34E-10	2.40E-07	7.99E-08	8.77E-07
GO:0005634~nucleus	152	1.94E-08	7.33E-06	1.83E-06	2.68E-05
GO:0005622~intracellular	312	2.48E-08	9.38E-06	1.88E-06	3.43E-05
GO:0043229~intracellular organelle	246	5.63E-08	2.13E-05	3.55E-06	7.79E-05
GO:0043226~organelle	246	6.45E-08	2.44E-05	3.48E-06	8.92E-05
GO:0044446~intracellular organelle part	154	7.12E-08	2.69E-05	3.36E-06	9.85E-05
GO:0044422~organelle part	154	7.70E-08	2.91E-05	3.23E-06	1.06E-04
GO:0044424~intracellular part	291	1.06E-07	4.00E-05	4.00E-06	1.46E-04
GO:0005654~nucleoplasm	36	3.85E-06	0.0014545	1.32E-04	0.0053268
GO:0005694~chromosome	39	5.60E-06	0.0021162	1.77E-04	0.0077524
GO:0044451~nucleoplasm part	32	2.21E-05	0.0083347	6.44E-04	0.0306246
GO:0000178~exosome (RNase complex)	7	2.65E-05	0.0099551	7.14E-04	0.0366074
GO:0000176~nuclear exosome (RNase complex)	7	2.65E-05	0.0099551	7.14E-04	0.0366074

This table contains only top 15 significant enriched terms from each of Biological Processes (BP), Molecular Function (MF) and Cellular Component (CC). Table includes GO term, count (number of genes in the list of significant genes with a given term), P value, Bonferroni, Benjamini and FDR values.

Results from DAVID and Blast2GO revealed that cellular processes and metabolic processes are significantly enriched terms. We next applied the DAVID functional annotation-clustering tool to genes involved in metabolic processes. The DAVID analysis yielded 34 significant enriched annotation clusters in our analysis (*p*<0.05) (S2 Table). These overrepresented clusters of GO terms co-associate with one another and similar annotations grouped together. This analysis revealed that a significant number of GO clusters (13 annotation clusters) were associated with epigenetic, transcription and gene expression related functions (Table 3). Examples include: regulation of transcription (enrichment score: 22.78), transcription (enrichment score: 20.06) as well as positive regulation of gene expression (enrichment score: 11.35) (Table 3). The genes related to nuclear chromatin (enrichment score: 4.06), chromatin modification, histone modifications and remodeling, and methyltransferase activity etc. (enrichment score: 3.68) were significantly enriched clusters (Table 3). These data suggest a general role for metabolic related genes of our resistant housefly transcriptome in regulation, transcription and gene expression that may enhance metabolic activity of resistant houseflies. Among metabolic related genes, our previously identified P450s were also included. These results prompt us to look into regulatory elements and CpG islands of P450s identified in the resistant housefly transcriptome.

**Table 3:** Functional annotation clusters of metabolic related genes involved in epigenetic, transcription and gene expression identified by DAVID in the transcriptome of insecticide resistant housefly.

Cluster	Category	GO terms	Enrichment Score	Count	*p*value	Fold enrichment
1	BP	GO:0045449~regulation of transcription	22.78	62	8.45E-34	5.7
1	BP	GO:0006355~regulation of transcription, DNA-dependent	22.78	50	5.44E-27	6
1	MF	GO:0030528~transcription regulator activity	22.78	45	1.00E-21	5.22
1	MF	GO:0003700~transcription factor activity	22.78	22	9.04E-09	4.47
2	BP	GO:0006350~transcription	20.06	35	4.05E-17	5.6
2	PIR	Transcription regulation	20.06	34	4.50E-25	10.65
2	PIR	Transcription	20.06	34	7.38E-25	10.49
3	BP	GO:0045941~positive regulation of transcription	11.35	16	2.90E-11	10.13
3	BP	GO:0010628~positive regulation of gene expression	11.35	16	3.29E-11	10.05
3	BP	GO:0045944~positive regulation of transcription from RNA polymerase II promoter	11.35	7	5.12E-05	10.28
3	MF	GO:0016563~transcription activator activity	11.35	15	5.87E-12	12.77
5	BP	GO:0045892~negative regulation of transcription, DNA-dependent	8.85	17	3.16E-09	6.68
5	BP	GO:0016481~negative regulation of transcription	8.85	17	1.37E-08	6.04
5	BP	GO:0000122~negative regulation of transcription from RNA polymerase II promoter	8.85	7	1.59E-04	8.43
5	MF	GO:0016564~transcription repressor activity	8.85	9	6.04E-05	6.53
10	BP	GO:0045944~positive regulation of transcription from RNA polymerase II promoter	3.78	7	5.12E-05	10.29
16	MF	GO:0008134~transcription factor binding	3.30	9	1.38E-05	8.01
16	MF	GO:0003712~transcription cofactor activity	3.30	6	0.001	7.54
16	MF	GO:0003713~transcription coactivator activity	3.30	4	0.008	9.60
18	BP	GO:0016441~posttranscriptional gene silencing	2.95	5	0.002	9.19
18	BP	GO:0035194~posttranscriptional gene silencing by RNA	2.95	5	0.002	9.19
24	MF	GO:0016566~specific transcriptional repressor activity	2.11	5	9.96E-04	11.0
29	BP	GO:0006366~transcription from RNA polymerase II promoter	1.82	6	0.01	4.45
29	BP	GO:0006351~transcription, DNA-dependent	1.82	6	0.034	3.32
29	CC	GO:0008023~transcription elongation factor complex	1.82	4	0.001	17.09
29	MF	GO:0003711~transcription elongation regulator activity	1.82	3	0.01	4.45
29	MF	GO:0016251~general RNA polymerase II transcription factor activity	1.82	5	0.025	4.45
13	BP	GO:0040029~regulation of gene expression, epigenetic	3.45	14	3.72E-07	6.09
13	BP	GO:0045814~negative regulation of gene expression, epigenetic	3.45	6	0.013	4.20
7	CC	GO:0000790~nuclear chromatin	4.06	7	9.98E-06	13.29
7	CC	GO:0000785~chromatin	4.06	10	2.87E-05	6.05
11	PIR	Chromatin regulator	3.68	10	2.17E-09	18.95
11	BP	GO:0016570~histone modification	3.68	7	8.84E-05	9.35
11		GO:0016571~histone methylation	3.68	4	0.003	4.14
11	BP	GO:0016568~chromatin modification	3.68	14	2.60E-09	9.19
11	BP	GO:0006325~chromatin organization	3.68	16	6.73E-09	6.92
11	BP	GO:0016569~covalent chromatin modification	3.68	7	8.84E-05	9.35
11	BP	GO:0006338~chromatin remodeling	3.68	6	3.58E04	9.59
11	MF	GO:0018024~histone-lysine N-methyltransferase activity	3.68	4	0.002	16.67
11	MF	GO:0042054~histone methyltransferase activity	3.68	4	0.002	15.08
11	MF	GO:0046974~histone methyltransferase activity (H3-K9 specific)	3.68	3	0.004	29.69
11	CC	GO:0035097~histone methyltransferase complex	3.68	3	0.007	22.79
11	CC	GO:0034708~methyltransferase complex	3.68	3	0.007	22.79
11	MF	GO:0016278~lysine N-methyltransferase activity	3.68	4	0.002	16.67
11	MF	GO:0016279~protein-lysine N-methyltransferase activity	3.68	4	0.002	16.67
11	MF	GO:0008276~protein methyltransferase activity	3.68	4	0.007	9.90
11	MF	GO:0008170~N-methyltransferase activity	3.68	4	0.009	9.05
13	BP	GO:0006342~chromatin silencing	3.45	6	0.013	4.20
11	BP	GO:0006479~protein amino acid methylation	3.68	4	0.006	10.50
11	BP	GO:0043414~biopolymer methylation	3.68	4	0.02	6.84
11	BP	GO:0032259~methylation	3.68	4	0.034	5.55
13	BP	GO:0016458~gene silencing	3.45	10	2.30E-04	4.74
18	BP	GO:0016441~posttranscriptional gene silencing	2.95	5	0.002	9.19
18	BP	GO:0035194~posttranscriptional gene silencing by RNA	2.95	5	0.002	9.19
18	BP	GO:0031047~gene silencing by RNA	2.95	5	0.003	8.17
34	BP	GO:0007307~eggshell chorion gene amplification	1.62	3	0.019	13.78

Functional annotation groups with geometric *p*-value less than 0.05 are listed. Class ontology: BP = biological processes, MF = molecular function, CC = cellular component, PIR = protein information resource. Count = Number of genes in ontology

### Functional classification of predicted proteins and P450s

**Fig 3:**
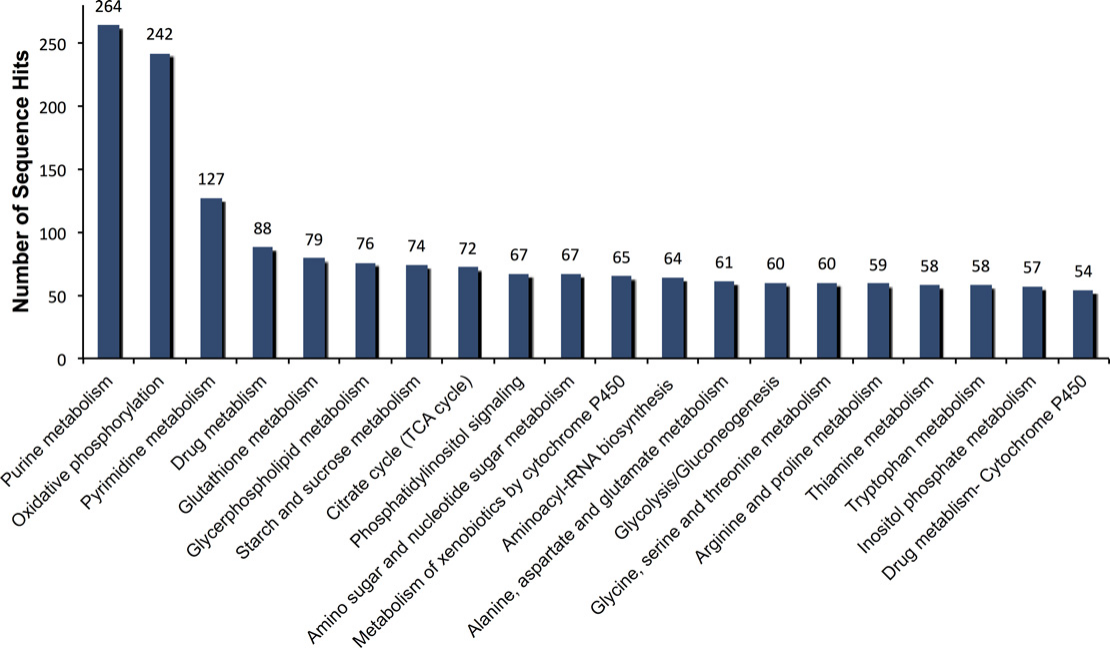
Distribution of contig sequences among KEGG (Kyoto Encyclopedia of Genes and Genome) pathways. The top 20 most highly represented pathways are shown. Analysis was performed using the Blast2GO and the KEGG database.

The Kyoto Encyclopedia of Genes and Genomes (KEGG) analysis was used to identify potential pathways represented in the transcriptome [46–48]. Among the annotated sequences, 3,648 sequences were annotated with an enzyme code EC number and mapped to 124 KEGG pathways. The distributions of the top 20 mapped KEGG pathways were included describing metabolic processes as the most represented pathways (Fig 3). In fact, the top five KEGG pathways were purine metabolism, oxidative phosphorylation, pyrimidine metabolism, drug metabolism and glutathione metabolism (Fig 3). Some sequences were mapped to several other pathways related to growth, reproduction and immunity such as cell cycle, CoA biosynthesis and inositol phosphate metabolism. The investment in metabolic transcripts may reflect maintenance of a high metabolic rate in houseflies. In our transcriptome of a spinosad-resistant strain, 120 contigs were annotated as P450s covering 44 P450s genes of *Musca domestica*. These include *cyp4g2*, *cyp6g4*, *cyp6a1*, *cyp6a36*, *cyp6a37*, *cyp6d1*, *cyp6d3* and *cyp12a2*. A recently published report found these P450s to be differentially expressed in a resistant housefly strain [29]. In view of significance of these P450s, we decided to check the presence of single nucleotide polymorphisms (SNPs).

### SNPs identification in differentially expressed P450s

In order to identify sequence variation associated with resistance to insecticides, sequenced fragments of aforementioned P450s from a resistant strain were compared with the susceptible reference strain WHO-SRS. In addition, we also included previously reported specific sequences of resistant strains to various insecticides from the USA [49–52]. The *cyp4g2* gene has three polymorphic sites at locations 67 (synonymous), 139 and 281 (non-synonymous) (Table 4). The SNPs at locations 67 and 281 were due to nucleotide substitutions, whereas there was an insertion of two nucleotides “AT” after location 139. The *cyp6a37* in resistant strains (791spin and a pyrethroid resistant strain from the USA) contain only one SNP at location 1102. It was a non-synonymous SNP that occur due to a nucleotide substitution, where the amino acid histidine (H) was converted to tyrosine (Y). Similarly, 8 SNPs were found in *cyp6d1* in the first exon at different locations. Among these SNPs, 7 were non-synonymous and only one was synonymous. The non-synonymous SNPs occur due to nucleotide substitution at location 1, 3, 10, 11, 13 and 14 whereas insertion of an additional cytosine occurs after the eleventh nucleotide. These substitutions and insertions resulted in conversion of amino acids where methionine (M) changes into leucine (L), glutamic acid (E) into serine (S) and leucine (L) into proline (P). Both insertion and substitution happened at location 11 resulting in conversion of the negatively charged glutamic acid (E) into the hydrophilic amino acid serine (S). There were also two SNPs found in *cyp12a2* at location 230 (non-synonymous) leading to conversion of lysine to methionine and at location 1114, is a synonymous SNP (Table 4).

**Table 4:** Differential expression, CpG islands, regulatory motifs and SNPs in selected P450s.

CYP P450	Copy number^1^	CpG island			Promoter Motif (PM)^2^	mRNA Motif (MM)^3^	SNPs		
		Frequency	Size (bp)	Location and coverage			Location	NA*	AA**
*CYP4G2*	2100±497 (2.2)	1	677	3’-end, covers exon 3	1, 3–6, 9, 13	1–3, 8, 13, 15, 16, 18, 19	67 (CTG), 281(GGT), 139^§^AT	C→T, G→T	L→L, G→V
*CYP6A1*	24.3±8.8 (2.7)	1	997	5’-end, covers exon 1 and promoter part	1–6, 8, 9, 11, 12	1–7, 9, 16			
*CYP6A36*	2.4±0.7 (0.4)	1	785	5’-end, exon 1 and promoter part	1, 3, 7, 8, 10	1–9, 11, 14			
*CYP6A37*	327±71.7 (2.8)	1	795	5’-end, exon 1 and promoter part	1, 5–7	1–7, 9, 11, 12, 15, 17, 20, 23	1102(CAT)	C→T	H→Y
*CYP6D1*	797±144 (0.7)	1	983	5’-end, covers all three exons, two introns and promoter part	2–6	1, 3, 4, 8, 10, 13, 23	1(ATG), 3(ATG), 9(GTA), 10(GAA), 11(GAA), 13(TTA), 14(TTA), 11^§^C	A→C, G→T, A→G, G →T, A→C, T→C, T→C,	M→L, M→L, V→V, E→S, E→S, L→P, L→P
*CYP6D3*	405±49.8 (5.8)	1	649	5’-end, covers 1st exon and intron	1–5, 7, 10–13	1, 3–5, 8–10, 12, 13, 18, 19, 21			
*CYP6G4*	363±95.5 (2.4)	1	850	Intergenic, covers exon 2	2, 3, 5, 7, 10, 12	1, 2, 4, 6, 7, 14, 17, 20–22			
*CYP12A2*	26.3±6.0 (0.7)	3	749, 675, 601	Intergenic and 3’-end, covers two introns and exon 3	3–5	3, 4, 6, 8, 13, 22	230(AAG), 1114(ACG)	A→T, G→A	K→M, T→T

^1^ Obtained from Højland *et al*. (2014) [29]

^2^ Promoter Motifs (PM) along with transcription factors with their significance can be found in Fig 4.

^3^ mRNA Motifs (MM) along with transcription factors can be found in Fig 5.

* Nucleic Acid

** Amino Acid

^§^ Insertion after specified nucleotide. Bold nucleotide letters represent site where mutation occur.

### Regulation of P450s

A comparative study of P450 gene expression in field and laboratory *Musca domestica* strains was conducted previously [42] and found massive changes in gene expression changes during adaptation to laboratory breeding of houseflies [42]. Based on our previous finding and the availability of the housefly genome [30] along with multiple *in silico* tools, we were challenged to get deeper into selected P450 genes to understand gene expression and regulation with their promoter analysis and CpG island detection.

#### Regulation of P450s; CpG islands

In our earlier report, it was demonstrated that *cyp6g4*, *cyp6a1* and *cyp6d3* were constitutively over-expressed in the resistant 791spin strain compared to the susceptible reference strain [29]. On the other hand, *cyp6a36*, *cyp6d1* and *cyp12a2* were highly expressed in the susceptible strain compared to the resistant strain and *cyp4g2* and *cyp6d1* have maximum basal expression compared to the other P450s (Table 4).

The P450s analyzed here, all contain a single CpG island site with the exception of *cyp12a2* that contains three CpG islands (Table 4). The location of the CpG island varies considerably among genes; *cyp6a1*, *cyp6a36*, *cyp6a37* and *cyp6d3* each contain a CpG island close to the 5´- region, *cyp4g2* and *cyp12a2* contain CpG islands close to their 3´region, whereas *cyp6g4* have a CpG island in the middle of the gene in an exon (Table 4). In our selected P450s, *cyp6a1* (997 bp), *cyp6d1* (983 bp) and *cyp6g4* (850 bp) contain a larger CpG island, whereas *cyp6d3* (649 bp), *cyp4g2* (677 bp) and *cyp12a2* (749 bp, 675 bp and 601 bp) contain comparatively shorter CpG islands. The CpG island of *cyp6d1* covers all three exons, two introns and part of the promoter region (Table 4).

#### Regulation of P450s; promoter motifs

**Fig 4:**
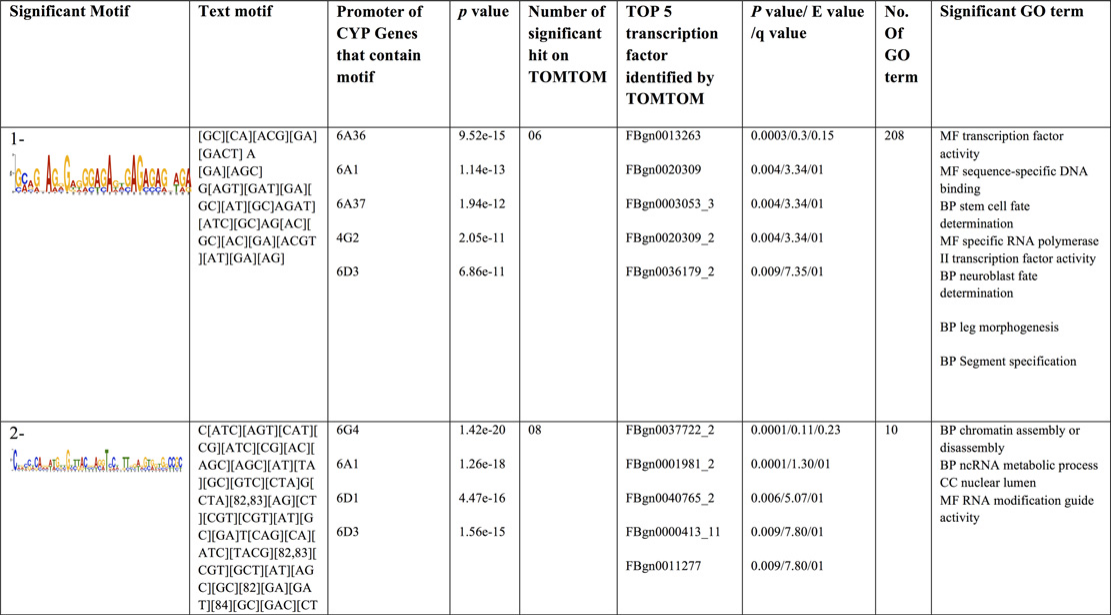
Motifs found in the 1000 bp upstream of promoter of selected CYPs P450 in the MEME analysis. The combination of TOMTOM and GOMO provides information regarding the novelty of the motif and the probability that this motif is involved in transcription regulation. The found motifs were presented with their e-value. GOMO is used to provide information on what type of GO term could be associated to this DNA motif using the *Drosophila melanogaster* sequence as reference. The TOMTOM hits provide information on the number of known transcription factor to which this motif is sequence wise close using Pearson correlation coefficient with E-value <10. The *p* value is the probability that the match occurred by random chance according to the null model, E value is the expected number of false positives in the matches up to this point and q value is the minimum **F**alse **D**iscovery **R**ate required to include the match.

Binding site motifs are important for regulatory function because they provide opportunity for a transcription factor to bind to genomic elements and hence affect the expression of nearby genes. In order to find such regulatory motifs in our selected P450s, we extracted the promoter and coding regions of relevant P450s from the susceptible housefly genome in search of potential regulatory motifs. In the MEME analysis, we identified 13 significant motifs in the promoter of selected P450s. The significant motifs (*p*-value <0.05) were presented with their logo and motif text (Fig 4). The motifs were numbered according to their *p*-values and top most number. The lower *p*-value means highest the significance. Amongst these promoter motifs, the most significant motif (number 1) was found in *cyp6a36*, *cyp6a1*, *cyp6a37*, *cyp4g2* and *cyp6d3*. The GOMO associates this motif to 208 GO terms and six transcription factors hit identified by TOMTOM (Fig 4). The most significant GO terms were stem cell fate determination, leg morphogenesis and segment specification in biological processes category, whereas in the molecular function category this motif associates with transcription factor activity, sequence specific DNA binding and specific RNA polymerase II transcription factor activity (Fig 4). The second significant motif was found in *cyp6g4*, *cyp6a1*, *cyp6d1* and *cyp6d3*. The GOMO associates it with eight GO terms and TOMTOM yields eight significant hit of known transcription factors. In the biological process GO terms, the most significant terms were chromatin assembly and disassembly and non-coding RNA metabolic processes, whereas the molecular function category contains RNA modification guide activity. The third motif was found in all eight P450s and involved in voltage gated calcium activity and DNA binding (Fig 4). The TOMTOM identified seven transcription factors for this motif. Similarly, the remaining ten motifs that were found in promoter regions of selected P450s were presented in Fig 4 along with their associate GO terms and transcription factors.

**Fig 5:**
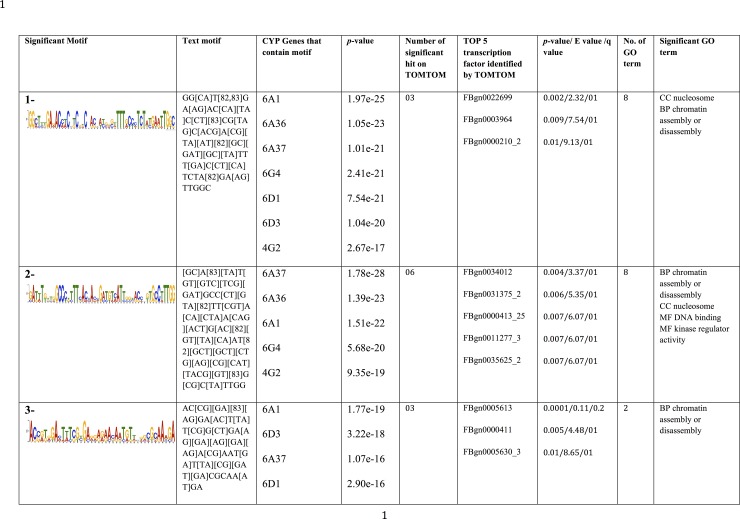
Motifs found in the mRNA of selected CYPs P450 in the MEME analysis. The combination of TOMTOM and GOMO provides information regarding the novelty of the motif and the probability that this motif is involved in transcription regulation. The found motifs were presented with their e-value. GOMO is used to provide information on what type of GO term could be associated to this DNA motif using the *Drosophila melanogaster* sequence as reference. The TOMTOM hits provide information on the number of known transcription factor to which this motif is sequence wise close Pearson correlation coefficient with E-value <10. The *p* value is the probability that the match occurred by random chance according to the null model, E value is the expected number of false positives in the matches up to this point and q value is the minimum **F**alse **D**iscovery **R**ate required to include the match.

The process of *cis*-regulatory elements discovery in mRNAs of selected P450s by MEME resulted in 23 significant motifs (Fig 5). In these mRNA motifs, chromatin assembly and disassembly were predominant GO terms that were present in 9 motifs. These motifs (motif 1, 2, 3, 4, 6, 14, 16, 20 and 22) were linked to chromatin assembly or disassembly. Some of these chromatin-linked motifs were present in specific P450s, but absent in the others (Fig 5). For example, motif 1 was present in all P450s except *cyp12a2*, motif 2 was present in *cyp6a37*, *cyp6a36*, *cyp6a1*, *cyp6g4* and *cyp4g2*, motif 3 in all, but *cyp6g4*, motif 4 in all but *cyp4g2* and so on (Fig 5). The numerous motifs such as motif 3, 8, 13, 15, 16 and 19 were present in *cyp4g2*, but absent in *cyp6g4*, whereas motifs 4, 6, 7, 14, 17, 20, 21 and 22 present in *cyp6g4*, but absent in *cyp4g2*. Similarly, several other motifs present in some specific P450s, but absent in others. All mRNA motifs were presented in Fig 5 along with their enriched GO terms and transcription factors. Some motif do not assign to any GO terms because GOMO did not find significant GO term in a *Drosophila* background.

## Discussion

In the context of investigating differential gene expression in a housefly population in adaptation to laboratory breeding [42], we used a housefly 454-transcriptome for annotation of transcripts. The transcriptome was based on mRNA from the 791spin strain, which is resistant to spinosad. We now dissect the 791spin transcriptome data to evaluate if we can get new information to understand spinosad resistance of this strain in general and the possible role of P450 enzymes in particular. Initially we describe and make an overview of the transcriptome. For this purpose Blast2GO, DAVID and KEGG tools were used for transcriptome analysis. The GO classification results are in line with recently sequenced and transcriptome analysis of houseflies [57–59] suggesting our transcriptome provide a comprehensive representation of *M*. *domestica*. Results from the DAVID tool and Blast2GO revealed that cellular processes and metabolic processes are significantly enriched terms. Moreover, functional annotation clustering analysis of genes involved in metabolic processes identified that a significant number of GO clusters related to epigenetic, gene expression and transcription. Furthermore, when assessing KEGG pathways a large number of contigs were also found to be associated with metabolism. The relative high investment in metabolic transcripts may reflect maintenance of high metabolic rate in the resistant housefly strain. One hundred and twenty contigs were annotated as P450s covering 44 different P450 genes of the housefly. It also include the eight differentially expressed P450 genes that we recently published [29]. In the housefly genome, there are 146 P450s genes along with 11 pseudogenes [28, 30]. Hence we only found one third of all the P450s, this could be due to lower sequence coverage and/or higher sequencing error rate of the 454 technology than Illumina RNAseq. These results were also in agreement with previous studies [60, 61]. However, 454-sequencing is more useful for resolving sequences with repetitive structures or metagenomic analysis in non-model organisms due to their longer read length [61]. The dataset of a resistant housefly strain generated by 454-seqeuncing can be useful to test sequence variations for SNPs identification due to its ability to generate longer read lengths.

The sequence variations of selected P450s in a resistant strain compared to a susceptible was conducted. For this purpose, we also included sequences of previously reported resistant strains to various insecticides from the USA in our study [49–52]. This single nucleotide polymorphism analysis resulted in identification of 12 SNPs due to nucleotide substitution and insertions. Out of 12 SNPs, eight were found in *cyp6d1*. In previous study conducted by Seifert and Scott G (2002) on a pyrethroid resistant housefly strain, five SNPs were reported. But in contrast to our identified SNPs, these SNPs were at different locations. Our identified SNPs occur at first 14 nucleotides of *cyp6d1*, whereas they found them at amino acid 150, 153, 165, 218, and 227 [62]. Moreover, our results also emphasized the significance of P450s in evolution of resistance in insects against pesticides. P450s can oxidize a wide range of xenobiotics and insects can become resistant to insecticides through these monooxygenases [6, 63]. The significance of P450s is magnified in metabolic based insecticide resistance scenario as these can detoxify even newly invented insecticides not marketed yet. Total P450s in housefly genomes were 157 including 11 pseudogenes and these numbers were significantly higher compared to other insects P450s [28, 30]. Higher numbers of P450s may suggest their role in houseflies for xenobiotic detoxification. The previous studies of 791spin spinosad resistance indicate that the resistance mechanism could be associated with P450 activity [27, 29]. This is also obvious from the fact that housefly is notorious to evolve resistance against several diverse toxins [14, 15, 19, 42, 51, 64].

Our functional annotation clustering of genes involved in metabolism revealed a significant number of GO clusters, which were related to transcription, gene expression, chromatin modification, histone modification and methyltransferase activity. These results encourage us to look into regulatory elements and CpG islands in the genes of our selected P450s. DNA methylation is an epigenetic marker of regulatory elements, which provide plasticity of gene expression. DNA methylation was initially not observed in *D*. *melanogaster* and other insects, but is now an establish fact now [36, 37, 65]. In insects methylated cytosines are primarily found in genes and, mounting evidence suggests that a specialized role exists for genic methylation in the regulation of transcription, and possibly mRNA splicing [66]. Recently methylation-changing agents were found to reduce sensitivity towards imidacloprid in *Aedes albopictus* in an epigenetic study [67]. Methylation reported to be specific to CpG islands in insects [38, 40], hence CpG islands in our selected P450s were explored. The CpG islands are CpG rich dinucleotide regions with a role in regulation and expression of a gene [68]. Active genes are generally unmethylated, but several studies reported that differential methylated regions were correlated with variable gene expression [69, 70]. The presence, location, frequency and length of CpG islands have functional consequences [68, 71–73]. For example, presence of CpG island in the promoter regions are the hallmark of widely expressed housekeeping genes [68]. On the other hand, the lack of CpG islands can result in DNA methylation leading to silencing of a particular gene. Recently, Krinner *et al*., (2014) found that CpG islands downstream of a transcription start site induce high levels of gene expression [74]. Similarly, the location of a CpG island decides the expression and regulation of a specific gene. It is well known that CpG dinucleotides situated in the 5′ region increase transgene expression to a greater extent compared to CpG dinucleotides in the 3′ region [74]. In our selected P450s only *cyp4g2* and *cyp12a2* contain CpG islands in their 3´region. Navin and Soojin (2011) demonstrated the functional relevance of CpG island length for regulation of gene expression [71]. They found that larger CpG islands are associated with tissue specific expression with a predominant role in environmental adaptations [71]. The *cyp6d1*, *cyp6a1* and *cyp6g4* have comparatively larger CpG islands and these P450s are well documented for their role in xenobiotic detoxification particularly in microevolution of insecticide resistance [29, 49, 52, 62]. However, further focused studies on CpG islands, their methylation pattern in resistant strains compared to susceptible are needed to draw any valid conclusion. Comprehensive investigations on CpG islands of P450s and other epigenetic modifications can provide insights into mechanisms and microevolution of regulatory complexity of gene expression. Our results just demonstrated the variations in length, location and frequency of CpG island of P450s to emphasize their potential significance that might determine specificity to perform specific functions through their expression or regulation. The CpG islands in P450s should be further studied as their regulation could provide insights how genotype and environment interact to determine insecticide resistance.

Regulatory elements or motifs are important molecular switches involved in the transcriptional regulation of a dynamic network of gene activities to determine functional specificity [75, 76]. In our analysis of selected P450s, some motifs were present in one specific P450, but absent in others. Hence, presence or absence of specific motifs might be essential to determine the specificity of those P450s. The motifs identified here were numbered according to their *p*-values. Lower *p*-value means higher significance and assigned top most number. The *p*-value is the probability that the match occurred by random chance according to the null model in an analyzed sequence of a gene. Moreover, in our analysis, the combination of TOMTOM and GOMO provides information regarding the novelty of the motif and the probability that this motif is involved in transcription regulation. Among the promoter motifs, the most significant motif was found in *cyp6a36*, *cyp6a1*, *cyp6a37*, *cyp4g2* and *cyp6d3* and the second most significant motif found in *cyp6g4*, *cyp6a1*, *cyp6d1* and *cyp6d3*. Similarly, among mRNA motifs, motif 1 is present in all P450s except *cyp12a2* and motif 2 is present in *cyp6a37*, *cyp6a36*, *cyp6a1*, *cyp6g4 and cyp4g2* and so on.

It’s well documented that the *cyp4g2* ortholog genes are conserved among all insect species and relates to cuticle morphogenesis, growth and several other cellular functions [77–80]. However, *cyp6g4* ortholog genes were not conserved and mostly involved in metabolic processes such as insecticide resistance [78–82]. In our motif analysis, these two genes (*cyp4g2* and *cyp6g4*) exhibited diversity in several motifs that were exclusively present in one, but absent in the other. For example, promoter motifs 1, 4, 6, 9 and 13 are present in *cyp4g2*, whereas promoter motifs 2, 7, 10 and 12 were present only in *cyp6g4*. Interestingly, motifs present only in *cyp4g2* gene were assigned to a wide range of GO terms in the GOMO analysis, whereas motifs present only in *cyp6g4* exhibited a less and more selective range of cellular processes. Some promoter motifs (3 and 5) were present in both genes and found to be involved in essential cellular processes such as voltage-gated calcium channel activity and RNA polymerase activity. Similarly, motif analysis of mRNA revealed the presence and absence of certain motifs in these two genes. We observed that motifs present in *cyp4g2* genes were involved in essential processes like cellular processes, whereas *cyp6g4* motifs were involved in various cellular processes including metabolic and catabolic processes. Interestingly, mRNA motifs related to GO terms associated with chromatin assembly or disassembly was higher in *cyp6g4* genes (five motifs) compared to *cyp4g2* (three motifs), whereas only one motif was common in both genes (Fig 5). The motifs presented in all P450s examined here, could be related to the general function of being a P450, whereas those present in specific P450s could be involved in the more specific function of those P450s. Overall, we provide information about regulatory elements of selected P450s in the context of housefly but not in the context of resistant strain 791spin. The reason to avoid regulatory elements in the context of resistant strain is due to lack of any experimental evidence. Several further experiments can be done to check any epigenetic modification in resistant strain compared to susceptible one. This could be achieved through testing methylation pattern of selected P450s. Similarly, histone modification can also be checked in resistant strain compared to susceptible strain. In order to investigate transcriptional regulation of selected P450s in resistant strain, common transcription factors involved in epigenetic interaction, cooperation among epigenetic components and chromatin remodeling factors can be tested experimentally through heterologous expression, protein purification, crystallography and homology modeling. Gene rearrangements of these selected P450s in resistant strain can also be tested in order to understand resistance mechanism and control of gene expression in resistant strain.

In conclusion, we presented a transcriptome analysis of an insecticide resistant housefly that helps us to identify specific transcripts rendering resistance in *M*. *domestica*. Here we not only present 791spin 454-transcriptome data that advance our knowledge about the resistance mechanism of 791spin, but also report information of selected P450s regarding SNPs, CpG islands, promoter motifs and their linked transcription factors that might be useful to understand P450 mediated insecticide resistance in *M*. *domestica*. Furthermore, functional annotation clustering of metabolic related genes and motif analysis of P450s revealed their association with epigenetic, transcription and gene expression related functions that may enhance metabolic activity of resistant houseflies. Overall, our findings will advance understanding of mechanism and role of P450s in insecticide detoxification.

## Supporting Information

S1 Fig Distribution of E value for annotated sequences. This represents the distribution of BLASTX homology search according to e-value range. BLAST analysis against the non-redundant database was performed with assembled contig sequences and e-value cut off 1e-5.
(TIF)

S2 Fig Species distribution of the contig sequences to the top BLAST hits. BLAST analysis against the non-redundant protein database was performed with an e-value cut off 1e-5.
(TIFF)

S1 Table Putative cytochrome P450 transcripts identified in resistant housefly transcriptome. (DOCX)

S2 Table Functional annotation clustering of identified metabolism related genes in the transcriptome of resistant housefly (Analyzed with the DAVID 6.7 BETA bioinformatic resource). (DOCX)
