# Supplementary figures and images for: Transcriptome Analysis of an Insecticide Resistant Housefly Strain: Insights about SNPs and Regulatory Elements in Cytochrome P450 Genes
Source: PLoS One. 2016 Mar 28;11(3):e0151434. doi: 10.1371/journal.pone.0151434 (PMC4809514; doi:10.1371/journal.pone.0151434)

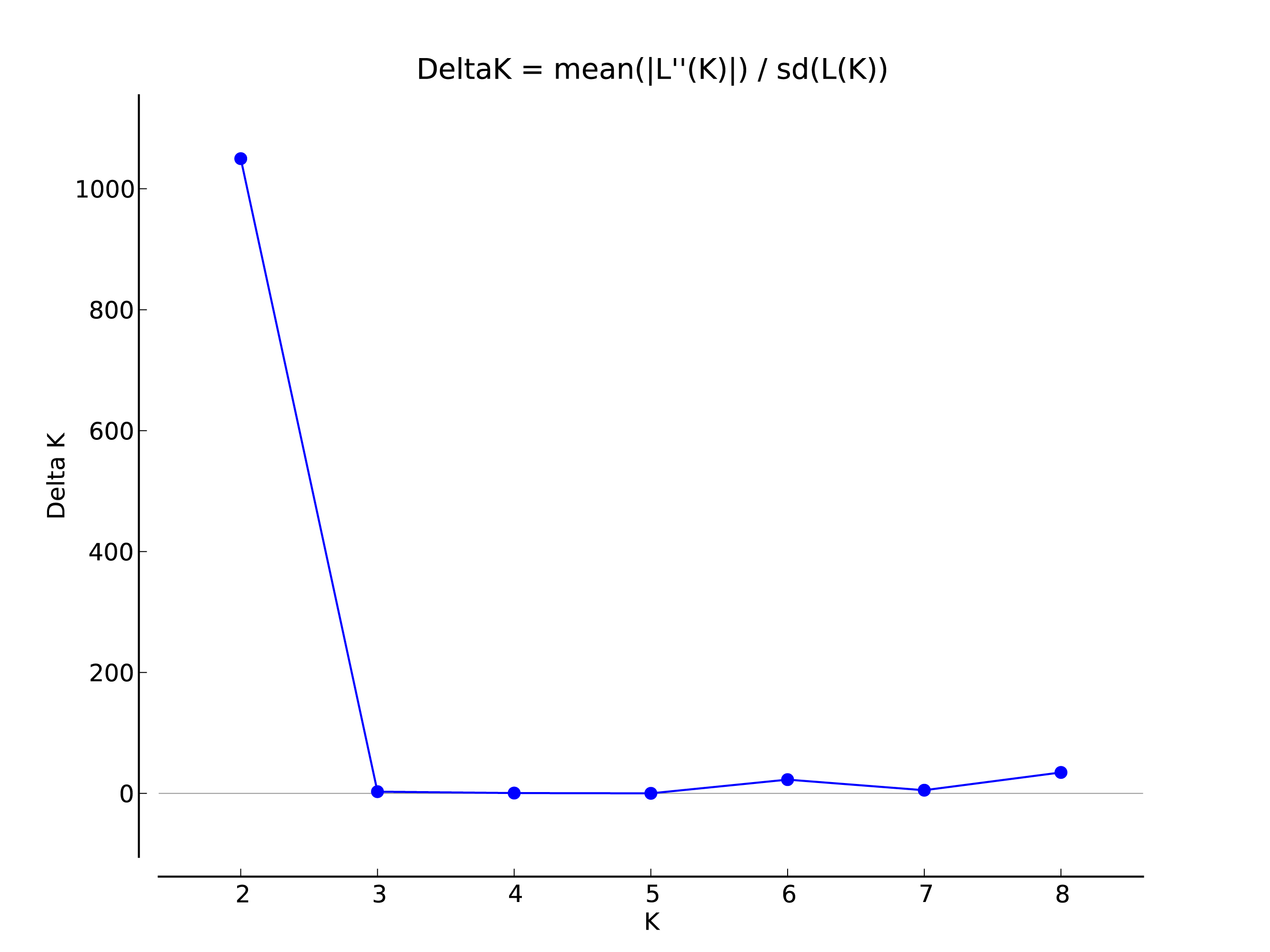

Supplement: S1 Fig — This represents the distribution of BLASTX homology search according to e-value range. BLAST analysis against the non-redundant database was performed with assembled contig sequences and e-value cut off 1e-5. (TIF) [file pone.0151434.s001.tif]

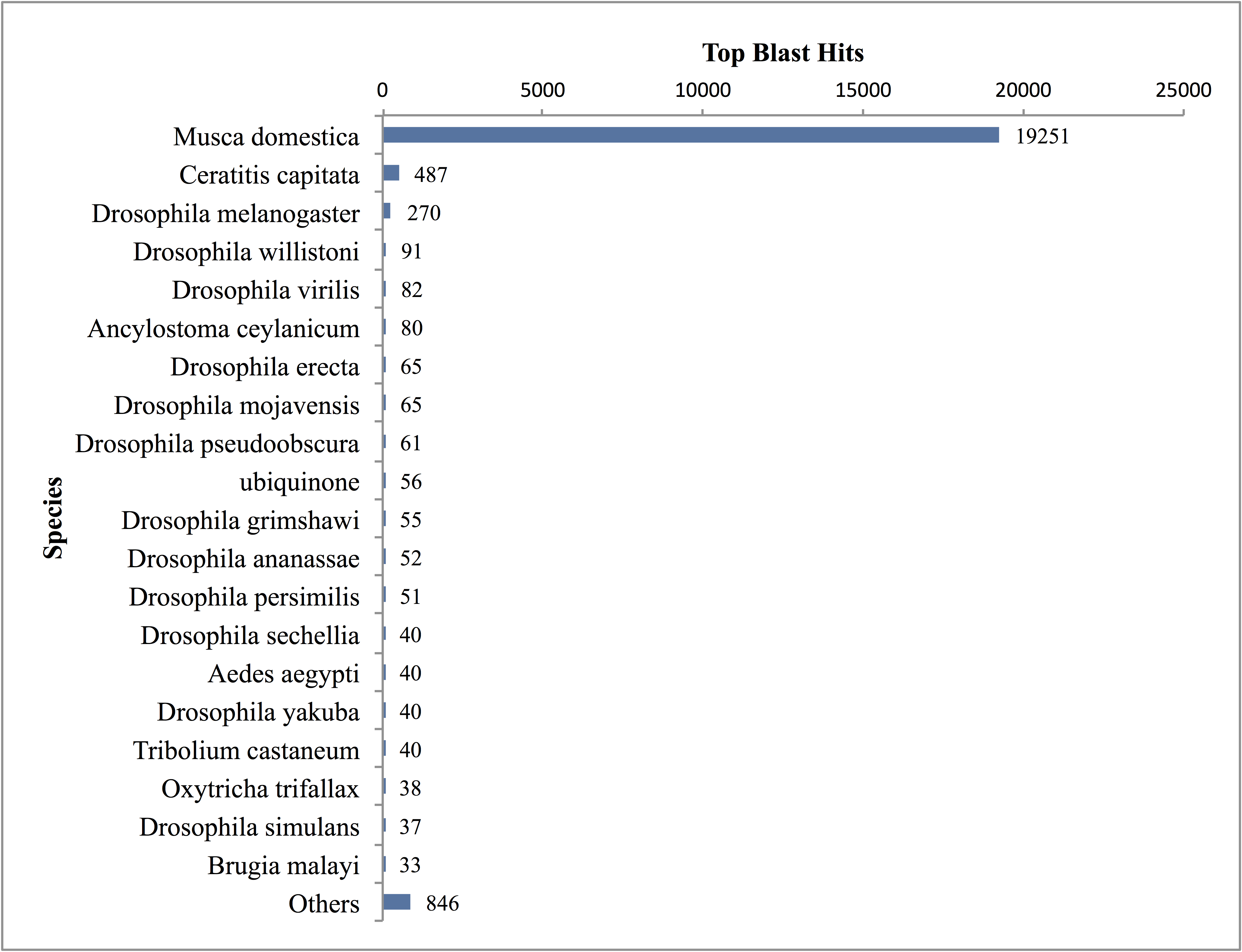

Supplement: S2 Fig — BLAST analysis against the non-redundant protein database was performed with an e-value cut off 1e-5. (TIFF) [file pone.0151434.s002.tiff]
