# Supplementary material for: Transcriptome Analysis of an Insecticide Resistant Housefly Strain: Insights about SNPs and Regulatory Elements in Cytochrome P450 Genes
Source: PLoS One. 2016 Mar 28;11(3):e0151434. doi: 10.1371/journal.pone.0151434 (PMC4809514; doi:10.1371/journal.pone.0151434)
Supplement: S1 Table — (DOCX) [file pone.0151434.s003.docx]

**S1 Table: Putative cytochrome P450 transcripts identified in resistant housefly transcriptome**

| **S.No.** | **Cytochrome P450** | **Locus number / Accession number** | **Contigs** | **No. of contigs** |
| --- | --- | --- | --- | --- |
| 1 | Cytochrome p450 6a22-like | LOC101893767/ [XM_005186997](http://www.ncbi.nlm.nih.gov/nuccore/XM_005186997.1) | M_dom_c2562 | 1 |
| 2 | Cytochrome p450 6a1-like | LOC101889365/ [NM_001287230.1](http://www.ncbi.nlm.nih.gov/nuccore/NM_001287230.1) | M_dom_rep_c19753, M_dom_rep_c8474, M_dom_rep_c7163, M_dom_rep_c34193 | 4 |
| 3 | Cytochrome p450 6d3-like | LOC101900431/ [XM_005184128.1](http://www.ncbi.nlm.nih.gov/nuccore/XM_005184128.1) | M_dom_rep_c6762, M_dom_rep_c12837 | 2 |
| 4 | Cytochrome p450 4ae1-like isoform x1 | LOC101898177/ [XM_005177253.1](http://www.ncbi.nlm.nih.gov/nuccore/XM_005177253.1) | M_dom_rep_c10079 | 1 |
| 5 | Cytochrome p450 4d1-like | LOC101898930/ [NM_001286903.1](http://www.ncbi.nlm.nih.gov/nuccore/NM_001286903.1) | M_dom_c146 | 1 |
| 6 | Cytochrome p450 4d2-like | LOC101897841/ [XM_005177251.1](http://www.ncbi.nlm.nih.gov/nuccore/XM_005177251.1) | M_dom_rep_c18385M_dom_c27668 | 2 |
| 7 | Cytochrome p450 4d8-like | LOC101888620/ [XM_005191906.1](http://www.ncbi.nlm.nih.gov/nuccore/XM_005191906.1) | M_dom_c27253, M_dom_c21960 | 2 |
| 8 | Cytochrome p450 4e2-like | LOC101896588/ [XM_005186271.1](http://www.ncbi.nlm.nih.gov/nuccore/XM_005186271.1) | M_dom_c4622 | 1 |
| 9 | Cytochrome p450 4g15-like | LOC101890640/ [XM_005177736.1](http://www.ncbi.nlm.nih.gov/nuccore/XM_005177736.1) | M_dom_c30350 | 1 |
| 10 | Cytochrome p450 4g1-like | LOC101887882/ [NM_001286897.1](http://www.ncbi.nlm.nih.gov/nuccore/NM_001286897.1) | M_dom_rep_c7817, M_dom_rep_c6971, M_dom_rep_c6003,M_dom_rep_c28044,M_dom_rep_c22765,M_dom_rep_c22714,M_dom_rep_c17586,M_dom_rep_c12426,M_dom_rep_c11387,M_dom_rep_c11288,M_dom_c1956, M_dom_rep_c30318,M_dom_rep_c17283,M_dom_rep_c13456 | 14 |
| 11 | Cytochrome p450 4p1-like | LOC101891224/ [XM_005180896.1](http://www.ncbi.nlm.nih.gov/nuccore/XM_005180896.1) | \| M_dom_rep_c8304, \| \| --- \| \| M_dom_rep_c22094, \| \| M_dom_c1618,  M_dom_rep_c12652 \| | 4 |
| 12 | Cytochrome p450 6a9-like | LOC101890373/ [XM_005184336.1](http://www.ncbi.nlm.nih.gov/nuccore/XM_005184336.1) | M_dom_rep_c1167,M_dom_c35216, M_dom_c29 | 3 |
| 13 | Cytochrome p450 6d1-like | LOC101900791/ [XM_005184130.1](http://www.ncbi.nlm.nih.gov/nuccore/XM_005184130.1) | M_dom_c33428,, M_dom_rep_c8528,M_dom_rep_c5625,M_dom_rep_c27233,M_dom_rep_c14635 | 5 |
| 14 | Cytochrome p450 6g1-like | LOC101898562/ [NM_001286882.1](http://www.ncbi.nlm.nih.gov/nuccore/NM_001286882.1) | M_dom_rep_c6373, M_dom_rep_c13526 | 2 |
| 15 | Cytochrome p450 cyp12a2-like | LOC101889684/ [XM_005179997.1](http://www.ncbi.nlm.nih.gov/nuccore/XM_005179997.1) | M_dom_rep_c2328,M_dom_c9347, M_dom_c5039, M_dom_c4957, M_dom_c26961, M_dom_c26394, M_dom_c23190, M_dom_c3951 | 8 |
| 16 | Cytochrome p450 cyp18a1 | LOC101900728/ [XM_005183375.1](http://www.ncbi.nlm.nih.gov/nuccore/XM_005183375.1) | M_dom_rep_c6849, | 1 |
| 17 | Cytochrome p450 cyp306a1 | LOC101901255/ [XM_005183378.1](http://www.ncbi.nlm.nih.gov/nuccore/XM_005183378.1) | M_dom_c34640, M_dom_c19876 | 2 |
| 18 | Cytochrome P450 reductase-like (CPR) | LOC101890161/ [NM_001286889.1](http://www.ncbi.nlm.nih.gov/nuccore/NM_001286889.1) | M_dom_rep_c23343,M_dom_rep_c16856,M_dom_rep_c16154,M_dom_rep_c14510 | 4 |
| 19 | Cytochrome P450 302a1, mitochondrial-like | LOC101888518/ [XM_005179206.1](http://www.ncbi.nlm.nih.gov/nuccore/XM_005179206.1) | M_dom_c4127 | 1 |
| 20 | nadph--cytochrome p450 reductase | LOC101890161/ [NM_001286889.1](http://www.ncbi.nlm.nih.gov/nuccore/NM_001286889.1) | M_dom_rep_c21012,M_dom_rep_c5530 | 2 |
| 21 | Cytochrome p450 28a5-like | LOC101900938/ [XM_005191969.1](http://www.ncbi.nlm.nih.gov/nuccore/XM_005191969.1) | M_dom_c27722, M_dom_c247 | 2 |
| 22 | Cytochrome p450 28d1-like | LOC101897279/ [NM_001286880.1](http://www.ncbi.nlm.nih.gov/nuccore/NM_001286880.1) | M_dom_rep_c32995M_dom_rep_c21570M_dom_c1608 | 3 |
| 23 | Cytochrome p450 304a1- like | LOC101895933/ [XM_005191871.1](http://www.ncbi.nlm.nih.gov/nuccore/XM_005191871.1) | M_dom_c2495, M_dom_c673, M_dom_c4527 | 3 |
| 24 | Cytochrome p450 305a1-like | LOC101898997/ [XM_005180589.1](http://www.ncbi.nlm.nih.gov/nuccore/XM_005180589.1) | M_dom_c24859 | 1 |
| 25 | Cytochrome p450 308a1-like | LOC101900906/ [XM_005183376.1](http://www.ncbi.nlm.nih.gov/nuccore/XM_005183376.1) | M_dom_rep_c31752M_dom_c2674, M_dom_rep_c7833, M_dom_rep_c27142 | 4 |
| 26 | Cytochrome p450 310a1-like | LOC101899919/ [XM_005184125.1](http://www.ncbi.nlm.nih.gov/nuccore/XM_005184125.1) | M_dom_rep_c29247 | 1 |
| 27 | Cytochrome p450 311a1-like | LOC101887394/ [XM_005180423.1](http://www.ncbi.nlm.nih.gov/nuccore/XM_005180423.1) | M_dom_rep_c20453 | 1 |
| 28 | Cytochrome p450 313a4-like | LOC101896830/ [XM_005187013.1](http://www.ncbi.nlm.nih.gov/nuccore/XM_005187013.1) | M_dom_rep_c8265, M_dom_rep_c7807,M_dom_c31653, M_dom_c30587, M_dom_c28384, M_dom_c1075 | 6 |
| 29 | Cytochrome p450 317a1-like | LOC101891061/ [XM_005184339.1](http://www.ncbi.nlm.nih.gov/nuccore/XM_005184339.1) | M_dom_c22837 | 1 |
| 30 | Cytochrome p450 4ac1-like | LOC101901643/ [XM_005186465.1](http://www.ncbi.nlm.nih.gov/nuccore/XM_005186465.1) | M_dom_rep_c23767M_dom_c19360 | 2 |
| 31 | Cytochrome p450 4d14-like | LOC101897209/ [XM_005177345.1](http://www.ncbi.nlm.nih.gov/nuccore/XM_005177345.1) | M_dom_c1971, M_dom_c19345, M_dom_c1893 | 3 |
| 32 | Cytochrome p450 4s3-like | LOC101891157/ [XM_005177488.1](http://www.ncbi.nlm.nih.gov/nuccore/XM_005177488.1) | M_dom_rep_c19873M_dom_c4836, M_dom_c2000 | 3 |
| 33 | Cytochrome p450 4aa1-like | LOC101895233/ [XM_005175977.1](http://www.ncbi.nlm.nih.gov/nuccore/XM_005175977.1) | M_dom_c32501 | 1 |
| 34 | Cytochrome p450 6a14-like | LOC101900065/ [XM_005175566.1](http://www.ncbi.nlm.nih.gov/nuccore/XM_005175566.1) | M_dom_rep_c7464, M_dom_rep_c9709,M_dom_rep_c23657 | 3 |
| 35 | Cytochrome p450 6a17-like | LOC101890543/ [XM_005184337.1](http://www.ncbi.nlm.nih.gov/nuccore/XM_005184337.1) | M_dom_rep_c24936 | 1 |
| 36 | Cytochrome p450 6a18-like | LOC101898668/ [XM_005185208.1](http://www.ncbi.nlm.nih.gov/nuccore/XM_005185208.1) | M_dom_c19100 | 1 |
| 37 | Cytochrome p450 6a21-like | LOC101889539/ [XM_005184332.1](http://www.ncbi.nlm.nih.gov/nuccore/XM_005184332.1) | M_dom_c3948, M_dom_c17998, M_dom_c25525, M_dom_rep_c10073,M_dom_rep_c26290 | 5 |
| 38 | Cytochrome p450 6d5-like | LOC101891297/ [NM_001286913.1](http://www.ncbi.nlm.nih.gov/nuccore/NM_001286913.1) | M_dom_rep_c12096,M_dom_c4916 | 2 |
| 39 | Cytochrome p450 6u1-like isoform x1 | LOC101887226/ [XM_005182161.1](http://www.ncbi.nlm.nih.gov/nuccore/XM_005182161.1) | M_dom_rep_c18147  M_dom_c24619, M_dom_rep_c17048 | 3 |
| 40 | Cytochrome p450 6v1-like | LOC101896204/ [XM_005176346.1](http://www.ncbi.nlm.nih.gov/nuccore/XM_005176346.1) | M_dom_c19497 | 1 |
| 41 | Cytochrome p450 9f2-like isoform x1 | LOC101898478/ [XM_005180050.1](http://www.ncbi.nlm.nih.gov/nuccore/XM_005180050.1) | M_dom_rep_c7313, M_dom_rep_c7214,M_dom_rep_c24890 | 3 |
| 42 | Probable cytochrome P450 12c1, mitochondrial-like | LOC101889539/ [XM_005184332.1](http://www.ncbi.nlm.nih.gov/nuccore/XM_005184332.1) | M_dom_c4504 | 1 |
| 43 | Cytochrome P450 315a1, mitochondrial-like | LOC101901025/ [XM_005184970.1](http://www.ncbi.nlm.nih.gov/nuccore/XM_005184970.1) | M_dom_c4504, M_dom_rep_c6152,M_dom_c30763, M_dom_c27358, M_dom_c11105, M_dom_rep_c28190 | 6 |
| 44 | Cytochrome p450 313b1 (pseudo) | LOC101895031/ | M_dom_rep_c21453 | 1 |
