# Supplementary material for: Transcriptome Analysis of an Insecticide Resistant Housefly Strain: Insights about SNPs and Regulatory Elements in Cytochrome P450 Genes
Source: PLoS One. 2016 Mar 28;11(3):e0151434. doi: 10.1371/journal.pone.0151434 (PMC4809514; doi:10.1371/journal.pone.0151434)
Supplement: S2 Table — (DOCX) [file pone.0151434.s004.docx]

**S2 Table: Functional annotation clustering of identified metabolism related genes in the transcriptome of resistant housefly** (Analyzed with the DAVID 6.7 BETA bioinformatic resource)

| **Cluster 1** | **Enrichment Score: 22.78** |  |  |  |  |
| --- | --- | --- | --- | --- | --- |
| Category | **Term** | **Count** | **PValue** | **FE** | **FDR** |
| BP | GO:0045449~regulation of transcription | 62 | 8.45E-34 | 5.7 | 1.34E-30 |
| BP | GO:0051252~regulation of RNA metabolic process | 57 | 2.87E-32 | 6.17 | 4.56E-29 |
| BP | GO:0006355~regulation of transcription, DNA-dependent | 50 | 5.44E-27 | 6 | 8.66E-24 |
| MF | GO:0030528~transcription regulator activity | 45 | 1.00E-21 | 5.22 | 1.23E-18 |
| MF | GO:0003677~DNA binding | 44 | 1.64E-17 | 4.19 | 2.02E-14 |
| MF | GO:0003700~transcription factor activity | 22 | 9.04E-09 | 4.47 | 1.11E-05 |
| **Cluster 2** | **Enrichment Score: 20.06** |  |  |  |  |
| PIR | Nucleus | 50 | 8.79E-30 | 6.97 | 9.76E-27 |
| PIR | Transcription regulation | 34 | 4.50E-25 | 10.65 | 4.99E-22 |
| PIR | Transcription | 34 | 7.38E-25 | 10.49 | 8.19E-22 |
| BP | GO:0006350~transcription | 35 | 4.05E-17 | 5.6 | 6.44E-14 |
| PIR | Activator | 16 | 1.41E-14 | 17.32 | 1.57E-11 |
| PIR | dna-binding | 24 | 2.41E-13 | 6.98 | 2.67E-10 |
| **Cluster 3** | **Enrichment Score: 11.35** |  |  |  |  |
| BP | GO:0009891~positive regulation of biosynthetic process | 22 | 5.46E-16 | 10.56 | 8.88E-13 |
| BP | GO:0031328~positive regulation of cellular biosynthetic process | 22 | 5.46E-16 | 10.56 | 8.88E-13 |
| BP | GO:0045935~positive regulation of nucleobase, nucleoside, nucleotide and nucleic acid metabolic process | 18 | 3.05E-13 | 10.93 | 4.86E-10 |
| BP | GO:0051173~positive regulation of nitrogen compound metabolic process | 18 | 3.05E-13 | 10.93 | 4.86E-10 |
| BP | GO:0010604~positive regulation of macromolecule metabolic process | 19 | 3.57E-13 | 9.83 | 5.67E-10 |
| BP | GO:0010557~positive regulation of macromolecule biosynthetic process | 18 | 8.97E-13 | 10.25 | 1.43E-09 |
| MF | GO:0016563~transcription activator activity | 15 | 5.87E-12 | 12.77 | 7.20E-09 |
| BP | GO:0045941~positive regulation of transcription | 16 | 2.90E-11 | 10.13 | 4.62E-08 |
| BP | GO:0010628~positive regulation of gene expression | 16 | 3.29E-11 | 10.05 | 5.24E-08 |
| BP | GO:0045893~positive regulation of transcription, DNA-dependent | 14 | 1.20E-10 | 11.69 | 1.91E-07 |
| BP | GO:0051254~positive regulation of RNA metabolic process | 14 | 1.61E-10 | 11.43 | 2.55E-07 |
| BP | GO:0045944~positive regulation of transcription from RNA polymerase II promoter | 7 | 5.12E-05 | 10.28 | 0.08 |
| **Cluster 4** | **Enrichment Score: 10.31** |  |  |  |  |
| CC | GO:0005654~nucleoplasm | 26 | 5.88E-15 | 6.73 | 7.03E-12 |
| CC | GO:0044451~nucleoplasm part | 24 | 6.94E-14 | 6.83 | 8.29E-11 |
| CC | GO:0031981~nuclear lumen | 27 | 4.85E-12 | 4.77 | 5.79E-09 |
| CC | GO:0070013~intracellular organelle lumen | 27 | 1.56E-08 | 3.32 | 1.86E-05 |
| CC | GO:0043233~organelle lumen | 27 | 1.56E-08 | 3.32 | 1.86E-05 |
| CC | GO:0031974~membrane-enclosed lumen | 27 | 2.73E-08 | 3.23 | 3.26E-05 |
| **Cluster 5** | **Enrichment Score: 8.85** |  |  |  |  |
| BP | GO:0010605~negative regulation of macromolecule metabolic process | 27 | 4.41E-14 | 6.25 | 7.01E-11 |
| BP | GO:0031327~negative regulation of cellular biosynthetic process | 22 | 1.03E-11 | 6.49 | 1.64E-08 |
| BP | GO:0009890~negative regulation of biosynthetic process | 22 | 1.03E-11 | 6.49 | 1.64E-08 |
| BP | GO:0010558~negative regulation of macromolecule biosynthetic process | 21 | 6.70E-11 | 6.27 | 1.07E-07 |
| BP | GO:0010629~negative regulation of gene expression | 21 | 1.38E-10 | 6.03 | 2.19E-07 |
| BP | GO:0045934~negative regulation of nucleobase, nucleoside, nucleotide and nucleic acid metabolic process | 19 | 7.60E-10 | 6.26 | 1.21E-06 |
| BP | GO:0051172~negative regulation of nitrogen compound metabolic process | 19 | 7.60E-10 | 6.26 | 1.21E-06 |
| BP | GO:0045892~negative regulation of transcription, DNA-dependent | 17 | 3.16E-09 | 6.68 | 5.02E-06 |
| BP | GO:0051253~negative regulation of RNA metabolic process | 17 | 5.00E-09 | 6.47 | 7.95E-06 |
| BP | GO:0016481~negative regulation of transcription | 17 | 1.37E-08 | 6.04 | 2.18E-05 |
| MF | GO:0016564~transcription repressor activity | 9 | 6.04E-05 | 6.53 | 0.07 |
| BP | GO:0000122~negative regulation of transcription from RNA polymerase II promoter | 7 | 1.59E-04 | 8.43 | 0.25 |
| **Cluster 6** | **Enrichment Score: 5.58** |  |  |  |  |
| BP | GO:0048477~oogenesis | 26 | 3.11E-08 | 3.54 | 4.94E-05 |
| BP | GO:0007292~female gamete generation | 26 | 4.01E-08 | 3.49 | 6.39E-05 |
| BP | GO:0007276~gamete generation | 28 | 5.43E-07 | 2.87 | 8.63E-04 |
| BP | GO:0019953~sexual reproduction | 28 | 9.38E-07 | 2.79 | 0.001 |
| BP | GO:0032504~multicellular organism reproduction | 28 | 2.84E-06 | 2.64 | 0.005 |
| BP | GO:0048609~reproductive process in a multicellular organism | 28 | 2.84E-06 | 2.64 | 0.005 |
| BP | GO:0048610~reproductive cellular process | 18 | 3.43E-04 | 2.64 | 0.544 |
| BP | GO:0003006~reproductive developmental process | 17 | 0.001 | 2.46 | 1.799 |
| **Cluster 7** | **Enrichment Score: 4.06** |  |  |  |  |
| CC | GO:0005694~chromosome | 20 | 2.60E-08 | 4.49 | 3.11E-05 |
| CC | GO:0044427~chromosomal part | 16 | 1.21E-06 | 4.5 | 0.001 |
| CC | GO:0005700~polytene chromosome | 10 | 5.30E-06 | 7.43 | 0.006 |
| CC | GO:0000790~nuclear chromatin | 7 | 9.98E-06 | 13.29 | 0.012 |
| CC | GO:0000785~chromatin | 10 | 2.87E-05 | 6.05 | 0.034 |
| CC | GO:0044454~nuclear chromosome part | 7 | 4.23E-04 | 6.93 | 0.504 |
| CC | GO:0000228~nuclear chromosome | 7 | 7.64E-04 | 6.21 | 0.908 |
| CC | GO:0031519~PcG protein complex | 4 | 9.32E-04 | 19.53 | 1.107 |
| CC | GO:0043232~intracellular non-membrane-bounded organelle | 21 | 0.040 | 1.52 | 38.863 |
| CC | GO:0043228~non-membrane-bounded organelle | 21 | 0.040 | 1.52 | 38.863 |
| **Cluster 8** | **Enrichment Score: 4.01** |  |  |  |  |
| BP | GO:0030182~neuron differentiation | 22 | 8.79E-08 | 3.95 | 1.40E-04 |
| BP | GO:0048666~neuron development | 17 | 1.44E-05 | 3.6 | 0.023 |
| BP | GO:0048812~neuron projection morphogenesis | 15 | 2.70E-05 | 3.85 | 0.043 |
| BP | GO:0031175~neuron projection development | 15 | 2.81E-05 | 3.84 | 0.045 |
| BP | GO:0048667~cell morphogenesis involved in neuron differentiation | 15 | 2.92E-05 | 3.82 | 0.046 |
| BP | GO:0000904~cell morphogenesis involved in differentiation | 15 | 5.10E-05 | 3.63 | 0.081 |
| BP | GO:0048858~cell projection morphogenesis | 15 | 9.21E-05 | 3.44 | 0.146 |
| BP | GO:0032990~cell part morphogenesis | 15 | 1.32E-04 | 3.33 | 0.210 |
| BP | GO:0030030~cell projection organization | 15 | 3.64E-04 | 3.02 | 0.577 |
| BP | GO:0032989~cellular component morphogenesis | 18 | 5.17E-04 | 2.55 | 0.819 |
| BP | GO:0000902~cell morphogenesis | 16 | 8.07E-04 | 2.66 | 1.275 |
| BP | GO:0016358~dendrite development | 8 | 9.77E-04 | 5.02 | 1.543 |
| BP | GO:0048813~dendrite morphogenesis | 8 | 9.77E-04 | 5.02 | 1.543 |
| BP | GO:0007517~muscle organ development | 8 | 0.002 | 4.19 | 4.285 |
| **Cluster 9** | **Enrichment Score: 3.84** |  |  |  |  |
| BP | GO:0070647~protein modification by small protein conjugation or removal | 9 | 2.83E-06 | 9.87 | 0.005 |
| BP | GO:0031647~regulation of protein stability | 6 | 3.02E-06 | 24.49 | 0.005 |
| PIR | Oogenesis | 7 | 5.39E-06 | 15.44 | 0.006 |
| BP | GO:0050821~protein stabilization | 5 | 1.42E-05 | 30.62 | 0.023 |
| PIR | signalosome | 4 | 2.88E-05 | 60.65 | 0.032 |
| MF | GO:0019781~NEDD8 activating enzyme activity | 4 | 1.01E-04 | 39.59 | 0.124 |
| BP | GO:0070646~protein modification by small protein removal | 5 | 1.04E-04 | 19.34 | 0.165 |
| BP | GO:0000338~protein deneddylation | 4 | 1.27E-04 | 36.75 | 0.202 |
| CC | GO:0008180~signalosome | 4 | 2.26E-04 | 30.39 | 0.270 |
| MF | GO:0008641~small protein activating enzyme activity | 4 | 3.84E-04 | 26.39 | 0.471 |
| INTERPRO | IPR000717: Proteasome component region PCI | 3 | 0.008 | 21.21 | 10.186 |
| SMART | SM00088: PINT | 3 | 0.021 | 13.08 | 19.525 |
| **Cluster 10** | **Enrichment Score: 3.78** |  |  |  |  |
| BP | GO:0051726~regulation of cell cycle | 17 | 5.42E-10 | 7.53 | 8.63E-07 |
| BP | GO:0007346~regulation of mitotic cell cycle | 10 | 6.08E-06 | 7.50 | 0.010 |
| BP | GO:0045787~positive regulation of cell cycle | 5 | 5.02E-05 | 22.97 | 0.080 |
| BP | GO:0045944~positive regulation of transcription from RNA polymerase II promoter | 7 | 5.12E-05 | 10.29 | 0.081 |
| BP | GO:0045931~positive regulation of mitotic cell cycle | 4 | 2.67E-04 | 29.40 | 0.424 |
| BP | GO:0010564~regulation of cell cycle process | 7 | 9.05E-04 | 6.12 | 1.429 |
| BP | GO:0045750~positive regulation of S phase of mitotic cell cycle | 3 | 0.004 | 31.50 | 5.602 |
| BP | GO:0007447~imaginal disc pattern formation | 6 | 0.009 | 4.59 | 13.940 |
| BP | GO:0007090~regulation of S phase of mitotic cell cycle | 3 | 0.013 | 16.96 | 18.460 |
| BP | GO:0033261~regulation of S phase | 3 | 0.015 | 15.75 | 21.042 |
| **Cluster 11** | **Enrichment Score: 3.68** |  |  |  |  |
| PIR | Chromatin regulator | 10 | 2.17E-09 | 18.95 | 2.41E-06 |
| BP | GO:0016568~chromatin modification | 14 | 2.60E-09 | 9.19 | 4.13E-06 |
| BP | GO:0051276~chromosome organization | 20 | 5.12E-09 | 5.21 | 8.15E-06 |
| BP | GO:0006325~chromatin organization | 16 | 6.73E-09 | 6.92 | 1.07E-05 |
| BP | GO:0016569~covalent chromatin modification | 7 | 8.84E-05 | 9.35 | 0.141 |
| BP | GO:0016570~histone modification | 7 | 8.84E-05 | 9.35 | 0.141 |
| BP | GO:0006338~chromatin remodeling | 6 | 3.58E-04 | 9.59 | 0.568 |
| MF | GO:0016278~lysine N-methyltransferase activity | 4 | 0.002 | 16.67 | 1.932 |
| MF | GO:0016279~protein-lysine N-methyltransferase activity | 4 | 0.002 | 16.67 | 1.932 |
| MF | GO:0018024~histone-lysine N-methyltransferase activity | 4 | 0.002 | 16.67 | 1.932 |
| MF | GO:0042054~histone methyltransferase activity | 4 | 0.002 | 15.08 | 2.596 |
| BP | GO:0016571~histone methylation | 4 | 0.003 | 14.00 | 4.141 |
| MF | GO:0046974~histone methyltransferase activity (H3-K9 specific) | 3 | 0.004 | 29.69 | 4.947 |
| BP | GO:0008213~protein amino acid alkylation | 4 | 0.006 | 10.50 | 9.286 |
| BP | GO:0006479~protein amino acid methylation | 4 | 0.006 | 10.50 | 9.286 |
| CC | GO:0035097~histone methyltransferase complex | 3 | 0.007 | 22.79 | 7.945 |
| CC | GO:0034708~methyltransferase complex | 3 | 0.007 | 22.79 | 7.945 |
| MF | GO:0008276~protein methyltransferase activity | 4 | 0.007 | 9.90 | 8.520 |
| MF | GO:0008170~N-methyltransferase activity | 4 | 0.009 | 9.05 | 10.819 |
| BP | GO:0043414~biopolymer methylation | 4 | 0.020 | 6.84 | 27.362 |
| BP | GO:0032259~methylation | 4 | 0.034 | 5.55 | 42.609 |
| **Cluster 12** | **Enrichment Score: 3.51** |  |  |  |  |
| BP | GO:0048024~regulation of nuclear mRNA splicing, via spliceosome | 7 | 1.90E-04 | 8.17 | 0.302 |
| BP | GO:0050684~regulation of mRNA processing | 7 | 1.90E-04 | 8.17 | 0.302 |
| BP | GO:0043484~regulation of RNA splicing | 7 | 2.46E-04 | 7.79 | 0.390 |
| BP | GO:0000381~regulation of alternative nuclear mRNA splicing, via spliceosome | 6 | 9.73E-04 | 7.74 | 1.536 |
| **Cluster 13** | **Enrichment Score: 3.45** |  |  |  |  |
| BP | GO:0040029~regulation of gene expression, epigenetic | 14 | 3.72E-07 | 6.09 | 5.91E-04 |
| BP | GO:0016458~gene silencing | 10 | 2.30E-04 | 4.74 | 0.365 |
| BP | GO:0006342~chromatin silencing | 6 | 0.013 | 4.20 | 19.441 |
| BP | GO:0045814~negative regulation of gene expression, epigenetic | 6 | 0.013 | 4.20 | 19.441 |
| **Cluster 14** | **Enrichment Score: 3.41** |  |  |  |  |
| BP | GO:0032268~regulation of cellular protein metabolic process | 12 | 1.21E-06 | 6.78 | 0.002 |
| BP | GO:0070647~protein modification by small protein conjugation or removal | 9 | 2.83E-06 | 9.87 | 0.005 |
| BP | GO:0009894~regulation of catabolic process | 5 | 1.28E-04 | 18.37 | 0.204 |
| BP | GO:0030162~regulation of proteolysis | 5 | 0.002 | 9.19 | 3.080 |
| BP | GO:0006417~regulation of translation | 6 | 0.003 | 6.12 | 4.318 |
| BP | GO:0032269~negative regulation of cellular protein metabolic process | 5 | 0.006 | 6.93 | 8.410 |
| BP | GO:0051248~negative regulation of protein metabolic process | 5 | 0.006 | 6.80 | 8.965 |
| BP | GO:0042176~regulation of protein catabolic process | 3 | 0.006 | 24.50 | 9.265 |
| **Cluster 15** | **Enrichment Score: 3.38** |  |  |  |  |
| BP | GO:0009894~regulation of catabolic process | 5 | 1.28E-04 | 18.37 | 0.20 |
| BP | GO:0031330~negative regulation of cellular catabolic process | 4 | 4.80E-04 | 24.50 | 0.76 |
| BP | GO:0009895~negative regulation of catabolic process | 4 | 6.18E-04 | 22.61 | 0.98 |
| BP | GO:0031329~regulation of cellular catabolic process | 4 | 7.78E-04 | 21.00 | 1.23 |
| **Cluster 16** | **Enrichment Score: 3.30** |  |  |  |  |
| MF | GO:0008134~transcription factor binding | 9 | 1.38E-05 | 8.01 | 0.017 |
| MF | GO:0003712~transcription cofactor activity | 6 | 0.001 | 7.54 | 1.333 |
| MF | GO:0003713~transcription coactivator activity | 4 | 0.008 | 9.60 | 9.255 |
| **Cluster 17** | **Enrichment Score: 3.14** |  |  |  |  |
| BP | GO:0019730~antimicrobial humoral response | 8 | 3.96E-05 | 8.40 | 0.063 |
| BP | GO:0006963~positive regulation of antibacterial peptide biosynthetic process | 5 | 8.26E-05 | 20.41 | 0.131 |
| BP | GO:0002786~regulation of antibacterial peptide production | 5 | 8.26E-05 | 20.41 | 0.131 |
| BP | GO:0002808~regulation of antibacterial peptide biosynthetic process | 5 | 8.26E-05 | 20.41 | 0.131 |
| BP | GO:0006959~humoral immune response | 8 | 1.19E-04 | 7.08 | 0.189 |
| BP | GO:0002697~regulation of immune effector process | 5 | 2.70E-04 | 15.31 | 0.428 |
| BP | GO:0002700~regulation of production of molecular mediator of immune response | 5 | 2.70E-04 | 15.31 | 0.428 |
| BP | GO:0002784~regulation of antimicrobial peptide production | 5 | 2.70E-04 | 15.31 | 0.428 |
| BP | GO:0002807~positive regulation of antimicrobial peptide biosynthetic process | 5 | 2.70E-04 | 15.31 | 0.428 |
| BP | GO:0002805~regulation of antimicrobial peptide biosynthetic process | 5 | 2.70E-04 | 15.31 | 0.428 |
| BP | GO:0006955~immune response | 10 | 4.36E-04 | 4.35 | 0.692 |
| BP | GO:0002759~regulation of antimicrobial humoral response | 5 | 4.98E-04 | 13.12 | 0.790 |
| BP | GO:0043900~regulation of multi-organism process | 5 | 4.98E-04 | 13.12 | 0.790 |
| BP | GO:0002831~regulation of response to biotic stimulus | 5 | 4.98E-04 | 13.12 | 0.790 |
| BP | GO:0002920~regulation of humoral immune response | 5 | 4.98E-04 | 13.12 | 0.790 |
| BP | GO:0008063~Toll signaling pathway | 4 | 0.007 | 9.80 | 11.178 |
| BP | GO:0006952~defense response | 8 | 0.009 | 3.40 | 12.959 |
| BP | GO:0042742~defense response to bacterium | 5 | 0.014 | 5.33 | 19.839 |
| BP | GO:0045088~regulation of innate immune response | 3 | 0.015 | 15.75 | 21.042 |
| BP | GO:0009617~response to bacterium | 5 | 0.023 | 4.59 | 30.467 |
| BP | GO:0045087~innate immune response | 5 | 0.035 | 3.99 | 43.578 |
| **Cluster 18** | **Enrichment Score: 2.95** |  |  |  |  |
| BP | GO:0016458~gene silencing | 10 | 2.30E-04 | 4.74 | 0.365 |
| BP | GO:0016246~RNA interference | 5 | 6.53E-04 | 12.25 | 1.034 |
| BP | GO:0016441~posttranscriptional gene silencing | 5 | 0.002 | 9.19 | 3.080 |
| BP | GO:0035194~posttranscriptional gene silencing by RNA | 5 | 0.002 | 9.19 | 3.080 |
| BP | GO:0031047~gene silencing by RNA | 5 | 0.003 | 8.17 | 4.731 |
| **Cluster 19** | **Enrichment Score: 2.92** |  |  |  |  |
| PIR | zinc-finger | 14 | 1.60E-06 | 5.41 | 0.002 |
| PIR | zinc | 17 | 1.69E-05 | 3.57 | 0.019 |
| PIR | metal-binding | 19 | 7.22E-05 | 2.90 | 0.080 |
| MF | GO:0008270~zinc ion binding | 24 | 0.003 | 1.85 | 4.049 |
| MF | GO:0046914~transition metal ion binding | 27 | 0.014 | 1.58 | 15.649 |
| MF | GO:0046872~metal ion binding | 31 | 0.028 | 1.43 | 29.245 |
| MF | GO:0043169~cation binding | 31 | 0.039 | 1.39 | 38.267 |
| MF | GO:0043167~ion binding | 31 | 0.041 | 1.39 | 39.947 |
| **Cluster 20** | **Enrichment Score: 2.81** |  |  |  |  |
| BP | GO:0045165~cell fate commitment | 12 | 2.75E-04 | 3.80 | 0.436 |
| BP | GO:0001709~cell fate determination | 9 | 3.32E-04 | 5.13 | 0.527 |
| BP | GO:0007422~peripheral nervous system development | 6 | 0.007 | 5.01 | 9.926 |
| BP | GO:0007423~sensory organ development | 13 | 0.009 | 2.33 | 13.255 |
| **Cluster 21** | **Enrichment Score: 2.48** |  |  |  |  |
| MF | GO:0004842~ubiquitin-protein ligase activity | 7 | 5.70E-04 | 6.68 | 0.697 |
| MF | GO:0019787~small conjugating protein ligase activity | 7 | 8.26E-04 | 6.23 | 1.009 |
| KEGG | Dme04120: Ubiquitin mediated proteolysis | 6 | 0.002 | 5.78 | 1.663 |
| MF | GO:0016881~acid-amino acid ligase activity | 7 | 0.003 | 4.86 | 3.562 |
| INTERPRO | IPR000608: Ubiquitin-conjugating enzyme, E2 | 4 | 0.003 | 13.20 | 4.082 |
| INTERPRO | IPR016135: Ubiquitin-conjugating enzyme/RWD-like | 4 | 0.005 | 11.31 | 6.281 |
| MF | GO:0016879~ligase activity, forming carbon-nitrogen bonds | 7 | 0.008 | 3.96 | 9.401 |
| PIR | ubl conjugation pathway | 5 | 0.008 | 6.19 | 8.977 |
| SMART | SM00212: UBCc | 4 | 0.012 | 8.14 | 11.837 |
| **Cluster 22** | **Enrichment Score: 2.41** |  |  |  |  |
| BP | GO:0046530~photoreceptor cell differentiation | 9 | 3.15E-04 | 5.17 | 0.500 |
| BP | GO:0001751~compound eye photoreceptor cell differentiation | 8 | 7.15E-04 | 5.30 | 1.131 |
| BP | GO:0001754~eye photoreceptor cell differentiation | 8 | 9.29E-04 | 5.07 | 1.467 |
| BP | GO:0007423~sensory organ development | 13 | 0.009 | 2.33 | 13.255 |
| BP | GO:0048592~eye morphogenesis | 10 | 0.010 | 2.73 | 15.068 |
| BP | GO:0001745~compound eye morphogenesis | 9 | 0.019 | 2.65 | 26.725 |
| BP | GO:0001654~eye development | 10 | 0.035 | 2.21 | 43.012 |
| **Cluster 23** | **Enrichment Score: 2.18** |  |  |  |  |
| PIR | Neurogenesis | 6 | 2.99E-04 | 10.11 | 0.332 |
| BP | GO:0007422~peripheral nervous system development | 6 | 0.007 | 5.01 | 9.926 |
| BP | GO:0008407~bristle morphogenesis | 4 | 0.025 | 6.25 | 33.318 |
| BP | GO:0022416~bristle development | 4 | 0.038 | 5.34 | 45.726 |
| **Cluster 24** | **Enrichment Score: 2.11** |  |  |  |  |
| MF | GO:0016566~specific transcriptional repressor activity | 5 | 9.96E-04 | 11.00 | 1.216 |
| BP | GO:0048645~organ formation | 5 | 0.002 | 9.19 | 3.080 |
| BP | GO:0007447~imaginal disc pattern formation | 6 | 0.009 | 4.59 | 13.940 |
| BP | GO:0010160~formation of organ boundary | 4 | 0.014 | 7.74 | 20.442 |
| BP | GO:0048859~formation of anatomical boundary | 4 | 0.021 | 6.68 | 28.823 |
| BP | GO:0035220~wing disc development | 9 | 0.036 | 2.35 | 43.801 |
| **Cluster 25** | **Enrichment Score: 1.97** |  |  |  |  |
| BP | GO:0043933~macromolecular complex subunit organization | 11 | 0.004 | 2.88 | 6.895 |
| BP | GO:0065003~macromolecular complex assembly | 10 | 0.006 | 2.99 | 8.870 |
| BP | GO:0022618~ribonucleoprotein complex assembly | 4 | 0.011 | 8.40 | 16.673 |
| BP | GO:0034621~cellular macromolecular complex subunit organization | 8 | 0.020 | 2.90 | 26.961 |
| BP | GO:0034622~cellular macromolecular complex assembly | 7 | 0.024 | 3.12 | 31.808 |
| **Cluster 26** | **Enrichment Score: 1.92** |  |  |  |  |
| BP | GO:0042981~regulation of apoptosis | 7 | 0.002 | 5.30 | 2.995 |
| BP | GO:0045476~nurse cell apoptosis | 3 | 0.004 | 31.50 | 5.602 |
| BP | GO:0043067~regulation of programmed cell death | 7 | 0.004 | 4.63 | 5.819 |
| BP | GO:0010941~regulation of cell death | 7 | 0.004 | 4.47 | 6.889 |
| BP | GO:0043065~positive regulation of apoptosis | 4 | 0.011 | 8.40 | 16.673 |
| BP | GO:0016567~protein ubiquitination | 4 | 0.019 | 7.00 | 25.924 |
| BP | GO:0008219~cell death | 7 | 0.022 | 3.18 | 29.754 |
| BP | GO:0043068~positive regulation of programmed cell death | 4 | 0.022 | 6.53 | 30.304 |
| BP | GO:0010942~positive regulation of cell death | 4 | 0.022 | 6.53 | 30.304 |
| BP | GO:0016265~death | 7 | 0.023 | 3.16 | 30.433 |
| BP | GO:0032446~protein modification by small protein conjugation | 4 | 0.027 | 6.12 | 34.847 |
| BP | GO:0006917~induction of apoptosis | 3 | 0.047 | 8.48 | 53.837 |
| **Cluster 27** | **Enrichment Score: 1.91** |  |  |  |  |
| BP | GO:0007444~imaginal disc development | 15 | 0.001 | 2.68 | 1.898 |
| BP | GO:0007389~pattern specification process | 16 | 0.002 | 2.45 | 2.901 |
| BP | GO:0048645~organ formation | 5 | 0.002 | 9.19 | 3.080 |
| BP | GO:0009791~post-embryonic development | 15 | 0.002 | 2.54 | 3.088 |
| BP | GO:0003002~regionalization | 15 | 0.003 | 2.43 | 4.642 |
| BP | GO:0002165~instar larval or pupal development | 14 | 0.004 | 2.46 | 6.065 |
| BP | GO:0035218~leg disc development | 5 | 0.005 | 7.20 | 7.364 |
| BP | GO:0007478~leg disc morphogenesis | 4 | 0.008 | 9.48 | 12.191 |
| BP | GO:0035282~segmentation | 10 | 0.008 | 2.83 | 12.373 |
| BP | GO:0035110~leg morphogenesis | 4 | 0.015 | 7.54 | 21.768 |
| BP | GO:0035107~appendage morphogenesis | 9 | 0.016 | 2.73 | 22.917 |
| BP | GO:0035108~limb morphogenesis | 4 | 0.016 | 7.35 | 23.125 |
| BP | GO:0060173~limb development | 4 | 0.016 | 7.35 | 23.125 |
| BP | GO:0048736~appendage development | 9 | 0.018 | 2.69 | 24.783 |
| BP | GO:0009886~post-embryonic morphogenesis | 11 | 0.020 | 2.29 | 27.883 |
| BP | GO:0007350~blastoderm segmentation | 8 | 0.028 | 2.70 | 35.911 |
| BP | GO:0035120~post-embryonic appendage morphogenesis | 8 | 0.035 | 2.57 | 43.008 |
| BP | GO:0009953~dorsal/ventral pattern formation | 6 | 0.035 | 3.27 | 43.646 |
| BP | GO:0035220~wing disc development | 9 | 0.036 | 2.35 | 43.801 |
| BP | GO:0009880~embryonic pattern specification | 8 | 0.036 | 2.55 | 44.327 |
| BP | GO:0035114~imaginal disc-derived appendage morphogenesis | 8 | 0.042 | 2.46 | 49.648 |
| BP | GO:0048707~instar larval or pupal morphogenesis | 10 | 0.044 | 2.12 | 51.156 |
| BP | GO:0048737~imaginal disc-derived appendage development | 8 | 0.045 | 2.42 | 52.313 |
| BP | GO:0048569~post-embryonic organ development | 9 | 0.047 | 2.23 | 53.346 |
| **Cluster 28** | **Enrichment Score: 1.84** |  |  |  |  |
| BP | GO:0010564~regulation of cell cycle process | 7 | 9.05E-04 | 6.12 | 1.429 |
| BP | GO:0030097~hemopoiesis | 4 | 0.020 | 6.84 | 27.362 |
| KEGG | Dme04330: Notch signaling pathway | 3 | 0.020 | 12.73 | 13.577 |
| BP | GO:0048534~hemopoietic or lymphoid organ development | 4 | 0.041 | 5.16 | 48.825 |
| BP | GO:0002520~immune system development | 4 | 0.041 | 5.16 | 48.825 |
| **Cluster 29** | **Enrichment Score: 1.82** |  |  |  |  |
| CC | GO:0008023~transcription elongation factor complex | 4 | 0.001 | 17.09 | 1.664 |
| BP | GO:0006354~RNA elongation | 3 | 0.009 | 20.04 | 13.600 |
| BP | GO:0006366~transcription from RNA polymerase II promoter | 6 | 0.011 | 4.45 | 15.660 |
| MF | GO:0003711~transcription elongation regulator activity | 3 | 0.015 | 15.84 | 16.530 |
| MF | GO:0016251~general RNA polymerase II transcription factor activity | 5 | 0.025 | 4.45 | 26.718 |
| BP | GO:0006351~transcription, DNA-dependent | 6 | 0.034 | 3.32 | 41.860 |
| BP | GO:0032774~RNA biosynthetic process | 6 | 0.036 | 3.24 | 44.541 |
| **Cluster 30** | **Enrichment Score: 1.81** |  |  |  |  |
| BP | GO:0048854~brain morphogenesis | 3 | 0.015 | 15.75 | 21.042 |
| BP | GO:0031987~locomotion involved in locomotory behavior | 3 | 0.015 | 15.75 | 21.042 |
| BP | GO:0001964~startle response | 3 | 0.015 | 15.75 | 21.042 |
| BP | GO:0050905~neuromuscular process | 3 | 0.017 | 14.70 | 23.701 |
| **Cluster 31** | **Enrichment Score: 1.80** |  |  |  |  |
| BP | GO:0006417~regulation of translation | 6 | 0.003 | 6.12 | 4.318 |
| MF | GO:0003743~translation initiation factor activity | 4 | 0.036 | 5.46 | 35.956 |
| BP | GO:0006413~translational initiation | 4 | 0.038 | 5.34 | 45.726 |
| Cluster 32 | **Enrichment Score: 1.74** |  |  |  |  |
| PIR | Helicase | 5 | 0.004 | 7.88 | 3.925 |
| MF | GO:0004386~helicase activity | 6 | 0.012 | 4.36 | 13.328 |
| MF | GO:0003678~DNA helicase activity | 4 | 0.013 | 7.92 | 15.262 |
| INTERPRO | IPR014021: Helicase, superfamily 1 and 2, ATP-binding | 4 | 0.037 | 5.42 | 37.959 |
| INTERPRO | IPR014001: DEAD-like helicase, N-terminal | 4 | 0.039 | 5.28 | 40.050 |
| INTERPRO | IPR001650: DNA/RNA helicase, C-terminal | 4 | 0.040 | 5.21 | 41.098 |
| **Cluster 33** | **Enrichment Score: 1.69** |  |  |  |  |
| PIR | atp-binding | 12 | 0.008 | 2.49 | 8.705 |
| PIR | Kinase | 6 | 0.030 | 3.43 | 28.530 |
| PIR | Serine/threonine-protein kinase | 5 | 0.033 | 4.10 | 31.083 |
| **Cluster 34** | **Enrichment Score: 1.64** |  |  |  |  |
| BP | GO:0006260~DNA replication | 6 | 0.009 | 4.59 | 13.940 |
| BP | GO:0007306~eggshell chorion assembly | 5 | 0.014 | 5.33 | 19.839 |
| BP | GO:0007307~eggshell chorion gene amplification | 3 | 0.019 | 13.78 | 26.422 |
| BP | GO:0030707~ovarian follicle cell development | 8 | 0.020 | 2.88 | 27.524 |
| BP | GO:0006277~DNA amplification | 3 | 0.027 | 11.60 | 34.789 |
| BP | GO:0010927~cellular component assembly involved in morphogenesis | 5 | 0.033 | 4.08 | 41.339 |
| BP | GO:0007304~chorion-containing eggshell formation | 5 | 0.038 | 3.91 | 45.824 |

FR= fold enrichment, Class ontology: BP= biological processes, MF= molecular function, CC= cellular component, PIR= protein information resource. Count= Number of genes in ontology.
